# The Gut Microbiome as a Mechanistic Link Between the Planetary Health Diet and Healthy Aging

**DOI:** 10.3390/nu18172864

**Published:** 2026-09-02

**Authors:** Tamás Jarecsny, Andrea Lehoczki, Tamás Csípő, Vince Fazekas-Pongor, Noémi Mózes, Boglárka Csík, Virág Zábó, Ágnes Lipécz, Dorottya Nyáry, Mónika Fekete

**Affiliations:** 1 Health Sciences Division, Doctoral School, Semmelweis University, 1085 Budapest, Hungary; jarecsny.tamas@janoskorhaz.hu (T.J.); ceglediandi@freemail.hu (A.L.); csipo.tamas@semmelweis.hu (T.C.); pongor.vince@semmelweis.hu (V.F.-P.); mozes.noemi@semmelweis.hu (N.M.); csik.boglarka@semmelweis.hu (B.C.); zabo.virag@semmelweis.hu (V.Z.); lipecz.agnes@semmelweis.hu (Á.L.); nyary.dorottya@stud.semmelweis.hu (D.N.); 2 Department of Neurology and Stroke, Saint John’s Central Hospital of North Buda, 1125 Budapest, Hungary; 3 Institute of Preventive Medicine and Public Health, Faculty of Medicine, Semmelweis University, 1085 Budapest, Hungary; 4 Fodor Center for Prevention and Healthy Aging, Semmelweis University, 1085 Budapest, Hungary

**Keywords:** Planetary Health Diet, gut microbiome, healthy aging, healthspan, geroscience, inflammaging, gut–brain axis, sustainable nutrition, environmental sustainability

## Abstract

Population aging, the rising burden of chronic non-communicable diseases, and environmental degradation are increasingly recognized as interconnected global challenges, underscoring the need for integrated strategies that simultaneously promote human and planetary health. The Planetary Health Diet (PHD), originally proposed to address these challenges, has emerged as a promising dietary framework for supporting healthy aging. Although its health benefits are increasingly supported by epidemiological and clinical evidence, the biological mechanisms underlying these effects remain incompletely understood. This narrative review synthesizes evidence on the interplay among the PHD, the gut microbiome, and biological aging, with emphasis on microbiome-mediated pathways connecting sustainable nutrition with healthspan. Adherence to PHD-consistent dietary patterns is associated with greater microbial diversity and functional capacity, increased production of bioactive microbial metabolites, and more favorable intestinal barrier, immune, and metabolic profiles. These adaptations may modulate several hallmarks of aging and contribute to the preservation of physiological resilience. Evidence from prospective cohort studies, randomized controlled trials, and systematic reviews further indicates that dietary patterns consistent with the PHD are associated with lower risks of cardiometabolic diseases, frailty, cognitive decline, and premature mortality. By integrating findings from nutritional science, microbiome research, geroscience, and public health, this review positions the gut microbiome as an important, although not exclusive, biological interface through which sustainable dietary patterns may support healthspan while contributing to environmental sustainability.

## 1. Introduction

Population aging, the increasing burden of chronic non-communicable diseases (NCDs), and accelerating environmental degradation have emerged as interconnected global challenges that require integrated preventive strategies [1]. Demographic shifts toward older populations have substantially increased the prevalence of multimorbidity, frailty, disability, and healthcare utilization, while unsustainable food systems simultaneously contribute to climate change, biodiversity loss, and resource depletion [2,3,4]. These parallel trends highlight the need for dietary approaches capable of improving both human and planetary health.

Healthy aging is increasingly defined by the preservation of functional capacity rather than the extension of lifespan alone. In the present review, healthy aging is distinguished from intermediate biological and disease-related outcomes. Direct healthy aging outcomes include measures of functional capacity, intrinsic capacity, frailty, disability-free or disease-free survival, healthspan, and longevity. In contrast, cardiometabolic disease risk, inflammatory and metabolic biomarkers, intestinal barrier function, microbial metabolites, and molecular signaling pathways are considered intermediate or mechanistically relevant outcomes rather than direct measures of healthy aging. From a geroscience perspective, prolonging healthspan requires maintaining metabolic and immune homeostasis while delaying the biological processes underlying age-related diseases [5]. Hallmarks of aging—including mitochondrial dysfunction, cellular senescence, genomic instability, epigenetic alterations, impaired proteostasis, inflammaging, and immunosenescence—form an interconnected biological network rather than independent pathological events [6,7,8].

Among the modifiable determinants of healthy aging, the gut microbiome has emerged as a key modulator of these processes [9]. Aging is consistently accompanied by reduced microbial diversity, depletion of beneficial short-chain fatty acid (SCFA)-producing bacteria such as *Faecalibacterium*, *Roseburia*, and *Bifidobacterium*, and expansion of pro-inflammatory taxa [10,11]. These alterations impair intestinal barrier integrity, increase microbial translocation, and promote chronic low-grade inflammation, thereby contributing to metabolic dysfunction, immune dysregulation, frailty, and cognitive decline [12]. Unlike many other hallmarks of aging, the gut microbiome remains highly responsive to dietary modification, making it an attractive target for preventive interventions [13].

Growing recognition of the interdependence between nutrition, environmental sustainability, and healthy aging has focused attention on the Planetary Health Diet (PHD), proposed by the EAT-Lancet Commission as a dietary model that simultaneously promotes human health and environmental sustainability [14,15]. The PHD emphasizes vegetables, fruits, legumes, whole grains, nuts, and unsaturated fats while limiting red meat, refined grains, and ultra-processed foods [16]. Large prospective cohort studies consistently associate greater adherence to these principles with lower risks of cardiovascular disease, type 2 diabetes, obesity, frailty, cognitive decline, and premature mortality [17,18,19,20].

Although these clinical benefits are increasingly recognized, the biological mechanisms underlying them remain incompletely understood. Existing reviews have largely examined sustainable nutrition, the gut microbiome, and healthy aging as separate research areas, with limited emphasis on the mechanistic pathways linking dietary exposures to the biology of aging [21,22].

Current evidence suggests that the gut microbiome represents an important, although not exclusive, biological interface through which PHD-consistent dietary patterns may influence host physiology. Fermentation of dietary fiber and microbial biotransformation of plant-derived phytochemicals generate bioactive metabolites—including SCFAs, secondary bile acids, indole derivatives, and polyphenol metabolites—that regulate nutrient-sensing pathways, immune function, mitochondrial homeostasis, and gut–brain communication [23,24,25,26]. These mechanisms provide a plausible biological framework linking sustainable dietary patterns with the preservation of physiological function during aging. They likely act in concert with other established nutritional and metabolic pathways that regulate the biology of aging.

**Figure 1:**
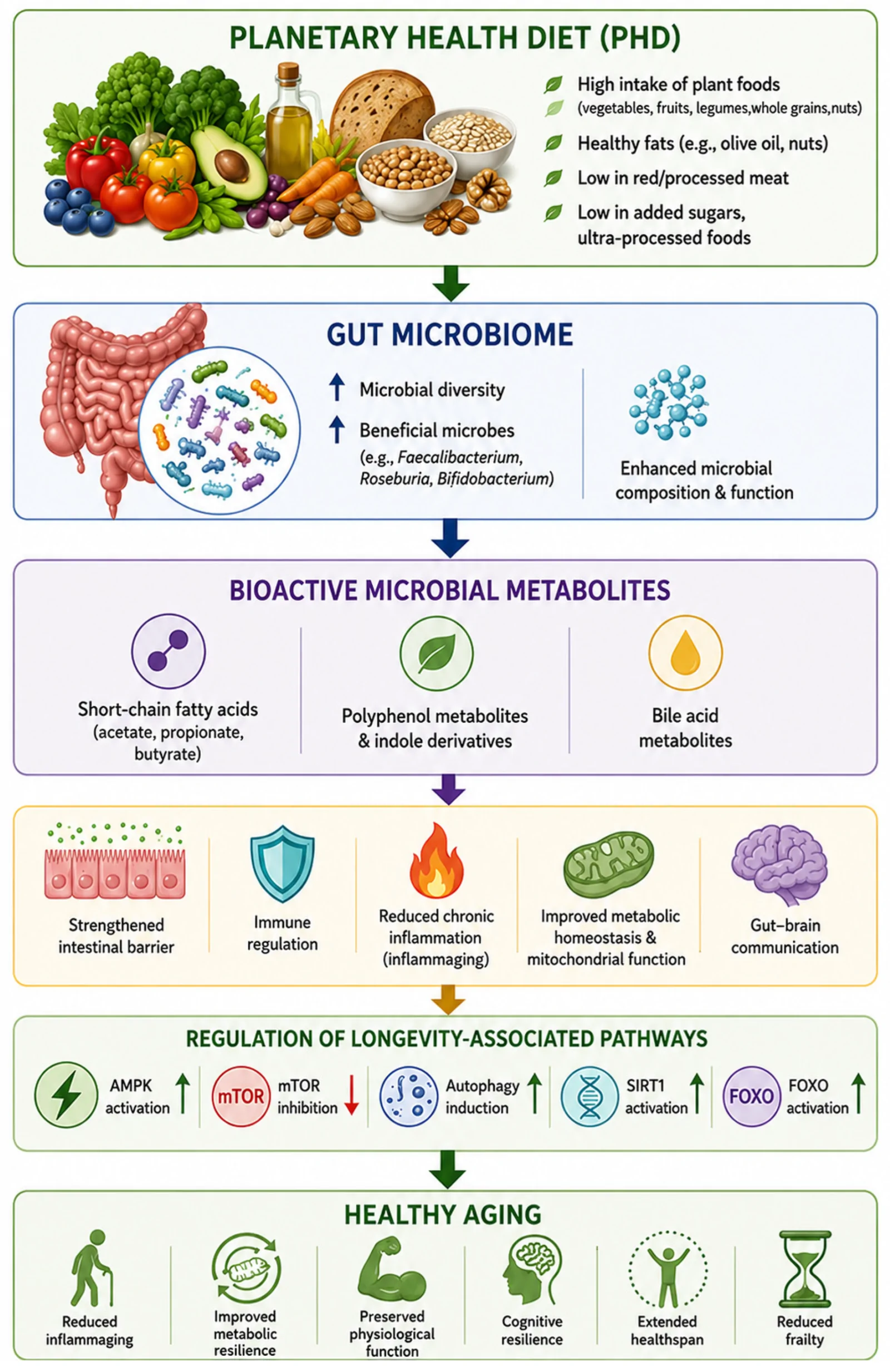
Proposed mechanistic framework linking the Planetary Health Diet to healthy aging through the gut microbiome. PHD-consistent dietary patterns may promote a diverse and metabolically active gut microbiome and increase the production of beneficial microbial metabolites, including short-chain fatty acids, bile acid metabolites, and polyphenol-derived metabolites. These metabolites may improve intestinal barrier integrity, regulate immune responses, reduce chronic inflammation, and influence longevity-related pathways, including AMPK, mTOR, autophagy, SIRT1, and FOXO. Through these mechanisms, the PHD–gut microbiome axis may support metabolic and physiological function, cognitive health, healthspan, and lower frailty risk. Arrows indicate the proposed direction of biological effects or pathway regulation; upward arrows indicate activation or increase, whereas downward arrows indicate inhibition or decrease. Several of these links are supported mainly by experimental or indirect human evidence rather than direct PHD-specific intervention studies. Abbreviations: PHD, Planetary Health Diet; AMPK, AMP-activated protein kinase; mTOR, mechanistic target of rapamycin; SIRT1, sirtuin 1; FOXO, forkhead box O.

This narrative review synthesizes current evidence on the role of the gut microbiome as a mechanistic link between the Planetary Health Diet and healthy aging. By integrating findings from nutritional science, microbiome research, geroscience, and public health, we describe how microbiome-mediated mechanisms interact with established physiological pathways to influence the hallmarks of aging, promote healthspan, and contribute to healthy aging (Figure 1).

## 2. Methods

### 2.1. Review Design

This narrative review provides an integrated overview of the mechanistic links between the Planetary Health Diet, the gut microbiome, and healthy aging. The review was informed by a structured and transparent literature search conducted in accordance with methodological recommendations for high-quality narrative reviews. Rather than quantitatively synthesizing the available evidence, the primary objective was to construct an evidence-based mechanistic framework describing how sustainable dietary patterns may influence healthspan through microbiome-mediated biological pathways.

### 2.2. Literature Search Strategy

The literature search was performed using PubMed/MEDLINE, Scopus, and the Web of Science Core Collection. Studies published between January 2005 and June 2026 were eligible for inclusion, with the final search conducted on 30 June 2026. This period was selected to capture the rapid expansion of gut microbiome research, major developments in longevity-associated signaling pathways, and the introduction of the Planetary Health Diet by the EAT-Lancet Commission in 2019. Earlier landmark studies relevant to diet, microbial metabolites, and healthy aging were also included where appropriate.

The search strategy combined Medical Subject Headings (MeSH) and free-text terms related to the Planetary Health Diet, sustainable nutrition, the gut microbiome, microbial metabolites, healthy aging, and longevity-associated pathways. Key terms included Planetary Health Diet, EAT-Lancet diet, sustainable nutrition, gut microbiome, gut microbiota, microbial metabolites, short-chain fatty acids, bile acids, tryptophan metabolites, healthy aging, healthspan, longevity, hallmarks of aging, inflammaging, immunosenescence, frailty, gut–brain axis, AMPK, mTOR, IGF-1, SIRT1, and autophagy, combined using the Boolean operators AND and OR. Reference lists of relevant reviews and original studies were also screened manually. Titles and abstracts were screened first, followed by full-text assessment of potentially eligible publications.

The database searches identified 6838 records. After duplicate removal, 2787 records underwent title and abstract screening, of which 420 publications were assessed in full text. A total of 350 publications were included in the final narrative synthesis. Because this review was designed as a narrative mechanistic synthesis, formal PRISMA-based study selection and quantitative evidence pooling were not undertaken. Additional landmark, methodological, and organizational sources were used to provide conceptual, definitional, and public health context but were not counted as part of the primary evidence synthesis. Study selection was conducted by members of the review team according to the predefined eligibility criteria.

### 2.3. Eligibility Criteria

Eligible publications included human studies, prospective cohort studies, randomized controlled trials, systematic reviews, meta-analyses, and mechanistic animal and in vitro studies with clear translational relevance that investigated relationships among the Planetary Health Diet, closely related plant-rich dietary patterns, the gut microbiome, microbial metabolites, and the molecular mechanisms of aging. Because direct microbiome studies of the PHD remain limited, evidence from closely related plant-rich dietary patterns was included when the relevant dietary components and proposed biological pathways substantially overlapped with those of the PHD. This evidence was considered supportive but indirect.

Particular emphasis was placed on studies examining interactions between microbial metabolites and host signaling pathways involved in longevity regulation, including AMPK, mTOR, IGF-1, SIRT1, FOXO, and autophagy, as well as studies addressing the hallmarks of aging, inflammaging, immunosenescence, frailty, cognitive decline, and cardiometabolic diseases. Conference abstracts, editorials, non-peer-reviewed publications, purely descriptive animal studies lacking translational relevance, and studies without clear mechanistic or clinical relevance were excluded.

### 2.4. Thematic Synthesis

The included studies were synthesized using a mechanism-oriented thematic approach. Evidence was organized according to predefined biological domains, including the effects of the Planetary Health Diet on gut microbial composition and function, microbial metabolites, intestinal barrier integrity, immune regulation, metabolic and neuroendocrine signaling, longevity-associated pathways, and the hallmarks of aging. Rather than summarizing individual studies sequentially, the evidence was integrated into a unified mechanistic framework describing how microbiome-mediated processes may influence cellular signaling, systemic inflammation, immune homeostasis, and the molecular biology of aging.

## 3. The Planetary Health Diet as a Microbiome-Modulating Dietary Pattern

Throughout Section 3, evidence from direct PHD interventions is distinguished from mechanistic evidence derived from individual PHD components and related plant-rich dietary patterns. The latter is considered supportive but indirect and should not be interpreted as evidence of PHD-specific effects.

### 3.1. Plant Diversity and Dietary Complexity

Plant diversity is one of the strongest dietary determinants of gut microbiome composition and function, exerting a greater influence than individual nutrients or single food groups [27]. Vegetables, fruits, whole grains, legumes, nuts, seeds, and herbs provide a broad range of fermentable fibers, resistant starches, and phytochemicals that create diverse ecological substrates for the gut microbiota, thereby promoting microbial diversity and functional capacity [28,29,30,31,32,33,34].

A defining feature of the Planetary Health Diet is its emphasis on consuming a wide variety of plant foods rather than simply reducing animal-source foods [35]. Greater dietary diversity may support microbial ecosystem resilience by promoting taxonomic and functional diversity and maintaining ecological stability in response to dietary and environmental perturbations [36,37].

Plant-rich dietary patterns are frequently associated with enrichment of several health-associated and fiber-fermenting microorganisms, including *Akkermansia muciniphila*, *Faecalibacterium prausnitzii*, *Roseburia* spp., *Bifidobacterium* spp., and members of the genus *Prevotella*, which are involved in mucin turnover, complex carbohydrate fermentation, and short-chain fatty acid production [38,39,40,41,42,43]. However, the physiological relevance of these taxa—particularly *Prevotella*—may vary according to species, strain, host characteristics, and dietary context. Human intervention studies investigating the PHD have reported modest taxonomic changes after relatively short intervention periods but consistently demonstrate enrichment of fiber-associated bacteria, suggesting that functional microbial adaptation may precede major compositional shifts [21,44].

Current evidence indicates that microbial function is a more informative indicator of dietary response than taxonomic composition alone [45]. By supplying structurally diverse fermentable substrates, the PHD enhances microbial metabolic activity and SCFA production, thereby supporting intestinal barrier integrity, immune homeostasis, and metabolic regulation [21,44,46,47].

### 3.2. Dietary Fiber and Microbial Fermentation

Dietary fiber is one of the principal characteristics of the PHD and represents its most important microbiome-modulating component [48]. Whole grains, legumes, vegetables, fruits, nuts, and seeds provide diverse fermentable substrates, including soluble and insoluble fibers, resistant starch, inulin, pectin, and β-glucans, which largely escape digestion in the upper gastrointestinal tract and undergo microbial fermentation in the colon [49,50].

This fermentation is primarily carried out by specialized fiber-degrading bacteria, including *Faecalibacterium*, *Roseburia*, *Eubacterium*, *Anaerostipes*, *Bifidobacterium*, and *Prevotella*, resulting in the production of SCFAs, predominantly acetate, propionate, and butyrate [51,52,53,54]. Although intervention studies evaluating the PHD have reported relatively modest taxonomic changes, they consistently demonstrate an increased abundance of fiber-fermenting bacteria, suggesting that dietary fiber primarily modulates microbial function rather than microbial composition [21,55].

SCFAs constitute the principal functional metabolites generated during microbial fermentation and are key mediators of host–microbiome communication [52]. Butyrate serves as the primary energy source for colonocytes and contributes to intestinal barrier integrity, whereas propionate and acetate regulate glucose, lipid, and whole-body energy metabolism [56,57,58].

The biological effects of SCFAs extend beyond the intestinal lumen through the regulation of conserved nutrient-sensing pathways involved in cellular homeostasis and healthy aging [24]. Consequently, the high fiber content of the PHD is considered a central driver of its microbiome-mediated health effects, providing the metabolic foundation for many of the beneficial effects of sustainable dietary patterns [59,60,61,62,63,64].

### 3.3. Polyphenols and Microbial Biotransformation

Polyphenols constitute another major microbiome-modulating component of the Planetary Health Diet. Vegetables, fruits, berries, legumes, whole grains, nuts, extra virgin olive oil, tea, and other plant-derived foods provide diverse classes of polyphenols, including flavonoids, phenolic acids, lignans, stilbenes, and ellagitannins [65,66]. Because many of these compounds are only partially absorbed in the small intestine, they reach the colon largely intact, where they undergo extensive microbial biotransformation [67,68].

The biological activity of dietary polyphenols depends largely on microbial metabolism rather than on the native compounds themselves [69]. Members of the genera *Gordonibacter*, *Eggerthella*, *Bifidobacterium*, *Lactobacillus*, *Eubacterium*, and *Clostridium* enzymatically convert complex polyphenols into smaller metabolites with greater bioavailability and biological activity [70,71,72,73]. Among the best-characterized products are urolithins derived from ellagitannins, together with several low-molecular-weight phenolic acids, including protocatechuic, ferulic, vanillic, and dihydrocaffeic acids [74,75].

Urolithin A has received particular attention because of its ability to promote mitophagy and improve mitochondrial quality control, effects associated with enhanced skeletal muscle function and metabolic resilience during aging [76,77,78,79]. Similarly, microbial phenolic metabolites interact with conserved longevity-associated signaling pathways, including SIRT1 and FOXO, thereby linking microbial metabolism with cellular adaptation to metabolic and oxidative stress [80,81,82,83,84].

Rather than acting as direct antioxidants, dietary polyphenols should be regarded as substrates for microbial bioactivation.

### 3.4. Legumes as Functional Prebiotic Foods

Legumes, including lentils, chickpeas, beans, and peas, are a fundamental component of the Planetary Health Diet and among its most potent microbiome-modulating foods [85]. In addition to their environmental sustainability, legumes provide a unique combination of fermentable dietary fiber, resistant starch, and raffinose-family oligosaccharides, including raffinose, stachyose, and verbascose, which resist digestion in the upper gastrointestinal tract and undergo microbial fermentation in the colon [86].

These substrates selectively stimulate the growth of fiber-fermenting bacteria, including *Faecalibacterium*, *Roseburia*, *Eubacterium*, and *Bifidobacterium*, thereby enhancing SCFA production and supporting microbial ecosystem stability [28,87].

Microbial fermentation of legumes also influences host metabolism through the production of SCFAs and the stimulation of enteroendocrine signaling. SCFAs promote glucagon-like peptide-1 (GLP-1) secretion, thereby linking microbial metabolism with improved glycemic regulation, insulin sensitivity, and appetite control [88,89,90,91,92,93,94,95,96].

### 3.5. Nuts and Seeds as Modulators of the Gut Microbiome and Bile Acid Metabolism

Nuts and seeds, including walnuts, almonds, hazelnuts, pistachios, pecans, cashews, flaxseed, chia seeds, and sunflower seeds, are integral components of the Planetary Health Diet. In addition to providing plant protein and dietary fiber, they are rich in monounsaturated and polyunsaturated fatty acids, phytosterols, tocopherols, and polyphenols, a combination consistently associated with reduced cardiometabolic risk in prospective cohort studies [19,97,98].

Beyond their established effects on blood lipid profiles, nuts and seeds influence the gut microbiome by promoting the growth of health-associated bacterial taxa, particularly *Akkermansia muciniphila* [38,99]. This mucin-degrading bacterium plays a key role in maintaining intestinal barrier integrity by preserving the mucus layer and epithelial function, thereby supporting gut homeostasis and limiting metabolic endotoxemia [100,101].

Diet-induced changes in microbial composition also influence bile acid metabolism. Intestinal microorganisms convert primary bile acids into a diverse pool of secondary bile acids that function as signaling molecules regulating glucose metabolism, lipid homeostasis, and immune responses through receptors such as the farnesoid X receptor (FXR) and the G protein-coupled bile acid receptor 1 (TGR5) [102,103,104,105,106,107,108].

### 3.6. Fermented Foods: Microbial Contributions to Immune Regulation and Intestinal Barrier Integrity

Although the PHD primarily emphasizes plant-based foods, fermented products such as yogurt with live cultures, kefir, sauerkraut, kimchi, and other traditionally fermented foods may further support gut microbial homeostasis [109]. These foods provide viable microorganisms together with fermentation-derived metabolites that influence microbial ecology, intestinal function, and host physiology [109,110,111].

Regular consumption of fermented foods has been associated with an increased abundance of beneficial genera, particularly *Lactobacillus* and *Bifidobacterium*, which contribute to colonization resistance and support a diverse microbial ecosystem [109].

A potential benefit of live microorganisms and fermentation-derived metabolites is their contribution to mucosal homeostasis. Their interactions with intestinal immune cells promote immune tolerance while reinforcing epithelial barrier function through enhanced mucin production, preservation of tight junction integrity, and stimulation of epithelial renewal [112,113,114,115,116,117,118].

### 3.7. Limiting Red and Processed Meat Intake: Reducing Pro-Inflammatory Microbial Metabolites

Limiting red and especially processed meat intake is a defining feature of the PHD [108]. Consistent with the EAT-Lancet recommendations, dietary protein is derived predominantly from legumes, nuts, and other plant foods and, to a lesser extent, from fish, dairy products, and poultry, whereas red meat consumption is substantially reduced [119,120].

Red meat is a major dietary source of L-carnitine and choline, which are converted by intestinal microorganisms into trimethylamine (TMA) and subsequently oxidized in the liver to trimethylamine *N*-oxide (TMAO) [121]. Elevated circulating TMAO concentrations have been consistently associated with increased risks of cardiovascular disease, chronic kidney disease, insulin resistance, and all-cause mortality, although the extent to which TMAO directly contributes to disease pathogenesis remains under investigation [121,122,123]. By reducing several dietary sources of TMA precursors, the PHD may decrease microbial TMA formation; however, circulating TMAO concentrations are also influenced by host metabolism, renal function, microbial composition, and other dietary sources.

Reduced consumption of red and processed meat also influences microbial bile acid metabolism. Diets rich in animal fat favor the formation of hydrophobic secondary bile acids, whereas the fiber-rich composition of the PHD promotes a bile acid profile associated with more favorable metabolic regulation [124,125]. Collectively, reduced red and processed meat intake may shift microbial metabolism away from pathways associated with TMA formation and unfavorable bile acid profiles, although the clinical relevance of these changes requires further confirmation in controlled human studies [126,127,128,129].

### 3.8. Healthy Fats: Modulation of the Gut Microbiome, Mitochondrial Function, and Membrane Integrity

Replacement of saturated animal fats with predominantly unsaturated plant-derived lipids is a defining characteristic of the PHD [65]. Extra virgin olive oil, nuts, seeds, and marine fish provide monounsaturated fatty acids (MUFAs), omega-3 polyunsaturated fatty acids (PUFAs), and a wide range of bioactive compounds consistently associated with improved cardiometabolic health and healthy aging [34,96,130,131].

In addition to their direct effects on host metabolism, unsaturated fatty acids influence gut microbial ecology. Diets rich in MUFAs, omega-3 PUFAs, and olive oil polyphenols consistently promote the enrichment of health-associated taxa, particularly *Akkermansia muciniphila*, a key regulator of intestinal barrier integrity and metabolic homeostasis [132,133].

Unsaturated fatty acids also contribute to cellular homeostasis through mechanisms involving both microbial metabolism and direct incorporation into cellular membranes. Incorporation of MUFAs and omega-3 PUFAs into membrane phospholipids preserves membrane fluidity and mitochondrial membrane integrity, while microbiome-derived metabolites further support metabolic flexibility and mitochondrial homeostasis [134,135,136] (Table 1).

## 4. The Gut Microbiome as an Important Modulator of Healthy Aging

### 4.1. Age-Related Changes in Gut Microbial Diversity

The gut microbiome undergoes substantial compositional and functional changes throughout aging, with accumulating evidence indicating that these alterations contribute to the biology of aging rather than merely reflecting chronological age [8,12,13]. Age-associated dysbiosis is characterized by declining microbial diversity, reduced functional capacity, and impaired ecosystem resilience, diminishing the ability of the gut microbiome to adapt to dietary changes, infections, medication use, and other environmental stressors [137,138].

A consistent feature of the aging microbiome is the depletion of several health-associated taxa involved in maintaining intestinal homeostasis. *Faecalibacterium prausnitzii*, *Roseburia* spp., and *Bifidobacterium* spp. are frequently depleted in frail, multimorbid, and institutionalized older adults, although age-related microbial patterns vary according to health status, geography, medication use, diet, and residential setting. Changes in *Akkermansia muciniphila* abundance are less consistent across populations [10,139,140]. These alterations are often accompanied by reduced microbial production of short-chain fatty acids, impaired bile acid metabolism, diminished vitamin biosynthesis, and weakened colonization resistance, reflecting a progressive loss of microbial functionality rather than taxonomic diversity alone.

In parallel, aging is frequently associated with expansion of opportunistic microorganisms, particularly members of the phylum *Proteobacteria*, together with disruption of intestinal barrier integrity and increased microbial translocation [140]. This imbalance promotes chronic low-grade inflammation and metabolic dysregulation, creating a biological environment that favors the development of multiple age-related disorders.

Growing evidence places age-related dysbiosis within the framework of geroscience as a modifiable mechanism influencing metabolic health, immune function, frailty, and neurodegeneration [8,141].

### 4.2. Gut Barrier Dysfunction as a Driver of Inflammaging

The intestinal barrier is a critical interface between the host and the gut microbiome, integrating epithelial integrity with immune surveillance and microbial containment. Beyond facilitating nutrient absorption, it prevents systemic exposure to luminal microorganisms and their products, thereby maintaining host–microbiome homeostasis. Progressive impairment of this barrier is increasingly recognized as an early event linking age-related dysbiosis with chronic systemic inflammation [142].

Aging compromises multiple structural components of the intestinal barrier. Reduced expression of tight junction proteins, including occludin, claudins, and zonula occludens-1 (ZO-1), together with diminished mucin production by goblet cells, weakens epithelial integrity and increases intestinal permeability [143]. This disruption, commonly referred to as “leaky gut,” is further promoted by dysregulated zonulin signaling and age-related alterations in microbial composition [144,145].

Loss of barrier integrity facilitates the translocation of microbial products, particularly lipopolysaccharide (LPS), into the systemic circulation [146]. Recognition of LPS by Toll-like receptor 4 (TLR4) initiates MyD88-dependent signaling, culminating in activation of nuclear factor kappa B (NF-κB), a central regulator of innate immune and inflammatory responses [147]. Sustained activation of this pathway promotes the production of pro-inflammatory mediators, including interleukin-1β, interleukin-6, tumor necrosis factor-α, and *C*-reactive protein, thereby reinforcing the chronic low-grade inflammatory state that characterizes aging [148].

Continuous microbial translocation amplifies immune activation, disrupts metabolic homeostasis, and contributes to several hallmarks of aging, including mitochondrial dysfunction, cellular senescence, and immunosenescence [149].

### 4.3. Microbial Metabolites: Short-Chain Fatty Acids as Longevity Signals

The biological effects of the gut microbiome are determined as much by its metabolic activity as by its taxonomic composition. Among microbiome-derived metabolites, SCFAs—primarily butyrate, propionate, and acetate—constitute the principal molecular mediators linking dietary fiber fermentation to host metabolism, immune regulation, and healthy aging [150,151].

Age-related dysbiosis is accompanied by a progressive decline in butyrate-producing bacteria, including *Faecalibacterium prausnitzii*, *Roseburia* spp., and other obligate anaerobes, resulting in reduced SCFA production [152]. This functional impairment compromises epithelial energy metabolism, weakens intestinal barrier integrity, and diminishes microbial regulation of systemic immune and metabolic homeostasis.

Among SCFAs, butyrate exerts the broadest range of geroprotective effects. As the primary energy source for colonocytes, it supports epithelial integrity while simultaneously functioning as an endogenous histone deacetylase inhibitor that modulates gene expression and inflammatory signaling [153]. Butyrate also promotes regulatory T-cell differentiation, enhances autophagy, preserves mitochondrial homeostasis, and activates AMPK, thereby coordinating multiple pathways involved in cellular maintenance and adaptive stress responses.

Propionate and acetate complement these actions through partially distinct physiological functions. Propionate contributes to glucose homeostasis by stimulating glucagon-like peptide-1 secretion and improving insulin sensitivity, whereas acetate serves as an important metabolic substrate and participates in appetite regulation, gut–brain communication, and neuroimmune signaling [152]. Although each SCFA exerts distinct biological effects, their coordinated actions integrate microbial metabolism with nutrient sensing, immune regulation, and tissue homeostasis.

### 4.4. Bile Acid Signaling and Host–Microbiome Crosstalk

Bile acids are now recognized as endocrine signaling molecules that mediate bidirectional communication between the gut microbiome and the host, extending far beyond their classical role in lipid digestion and the absorption of fat-soluble vitamins [154]. Following hepatic synthesis, primary bile acids are converted by the intestinal microbiome into secondary bile acids with distinct biological activities, thereby shaping receptor-mediated signaling pathways that regulate metabolism, immunity, and energy homeostasis [155].

A major mediator of these effects is the farnesoid X receptor (FXR), expressed predominantly in the liver and intestinal epithelium. FXR integrates bile acid metabolism with glucose and lipid homeostasis while restraining hepatic lipogenesis and inflammatory signaling. Age-associated dysbiosis alters the composition of the bile acid pool, leading to impaired FXR activation and disruption of metabolic homeostasis [156,157].

Complementary regulation is provided by the G protein-coupled bile acid receptor 1 (TGR5), which is expressed in immune cells, enteroendocrine cells, and brown adipose tissue. Activation of TGR5 enhances energy expenditure, stimulates glucagon-like peptide-1 secretion, improves insulin sensitivity, and attenuates macrophage-mediated inflammatory responses [158,159]. By supporting a microbial environment associated with more balanced bile acid metabolism, PHD-consistent dietary patterns may help maintain FXR- and TGR5-dependent signaling and related metabolic and immune functions [160,161].

### 4.5. Microbial Tryptophan Metabolism and the Gut–Brain–Immune Axis

Beyond short-chain fatty acids, microbial metabolism of dietary tryptophan represents another major pathway through which the gut microbiome influences immune function, neuroendocrine regulation, and healthy aging [162,163]. Through the production of bioactive indole metabolites, intestinal microorganisms provide a plausible molecular pathway linking dietary composition with host signaling networks involved in mucosal homeostasis and brain function.

Several commensal bacteria convert tryptophan into indole derivatives that serve as endogenous ligands for the aryl hydrocarbon receptor (AhR). Activation of AhR promotes epithelial regeneration, stimulates interleukin-22 production, enhances antimicrobial peptide expression, and preserves intestinal barrier integrity, thereby maintaining immune tolerance while limiting excessive inflammatory responses [164,165].

Age-related dysbiosis disrupts microbial tryptophan metabolism, resulting in reduced production of protective indole metabolites and impaired AhR signaling [166,167]. This functional impairment compromises mucosal homeostasis, promotes chronic low-grade inflammation, and contributes to the progressive decline in immune resilience observed during biological aging.

Microbial regulation of tryptophan metabolism also extends to neuroendocrine function. By influencing tryptophan availability and host serotonin biosynthesis, the gut microbiome modulates gut–brain communication, gastrointestinal physiology, and neuronal signaling [168,169]. Under chronic inflammatory conditions, however, tryptophan metabolism is increasingly redirected toward the kynurenine pathway through activation of indoleamine 2,3-dioxygenase (IDO). Persistent accumulation of kynurenine metabolites has been associated with neuroinflammation, cognitive impairment, depression, and immunosenescence, highlighting a shift from homeostatic to pro-inflammatory metabolism during aging [170,171].

### 4.6. Polyphenol-Derived Microbial Metabolites as Geroprotective Molecules

The biological activity of many dietary polyphenols depends on their transformation by the gut microbiome rather than on the parent compounds themselves. Because most flavonoids, ellagitannins, isoflavones, and other complex phytochemicals reach the colon largely intact, microbial enzymes convert them into low-molecular-weight metabolites with substantially greater bioavailability and biological activity [79,172].

Among these metabolites, urolithin A, generated from ellagitannins and ellagic acid by specific intestinal bacteria, has emerged as one of the best-characterized naturally occurring geroprotective molecules. Experimental and clinical studies demonstrate that urolithin A enhances mitophagy, improves mitochondrial quality control, and supports cellular energy metabolism, thereby targeting one of the central hallmarks of aging—mitochondrial dysfunction [173,174,175].

Equol, a microbial metabolite produced from soy isoflavones, provides another example of microbiome-dependent bioactivation. In addition to its antioxidant, anti-inflammatory, and estrogen receptor-modulating properties, equol production varies markedly among individuals because it depends on the presence of specific microbial communities [176]. This interindividual variability illustrates that the biological response to plant-rich diets is determined not only by dietary intake but also by the metabolic capacity of the gut microbiome.

Beyond urolithin A and equol, microbial metabolism generates numerous phenolic acids that regulate oxidative stress responses, inflammatory signaling, autophagy, and longevity-associated pathways, including SIRT1 and FOXO [177]. These metabolites integrate microbial metabolism with cellular stress adaptation and metabolic homeostasis, extending the biological effects of dietary polyphenols well beyond their intrinsic antioxidant capacity.

### 4.7. Microbial Endotoxins and Chronic Low-Grade Inflammation

Microbial endotoxemia represents an important mechanism through which age-related dysbiosis may contribute to chronic systemic inflammation. Among microbiome-derived inflammatory mediators, LPS, a structural component of the outer membrane of Gram-negative bacteria, has been most extensively implicated in the pathogenesis of inflammaging [178]. Following disruption of intestinal barrier integrity, increased systemic exposure to LPS initiates persistent innate immune activation that extends well beyond the gastrointestinal tract.

Recognition of circulating LPS by TLR4 activates MyD88-dependent signaling and the NF-κB pathway, resulting in sustained production of pro-inflammatory mediators, including interleukin-1β, interleukin-6, and tumor necrosis factor-α [178].

LPS also activates the NLRP3 inflammasome, thereby promoting maturation of interleukin-1β and interleukin-18 while amplifying sterile inflammatory responses [179]. Persistent inflammasome activation disrupts mitochondrial homeostasis, increases oxidative stress, and impairs cellular repair mechanisms, providing a mechanistic link between microbial endotoxemia and multiple hallmarks of aging.

Chronic inflammatory signaling further accelerates the accumulation of senescent cells and the development of the senescence-associated secretory phenotype (SASP). The resulting feed-forward cycle, in which microbial endotoxins, innate immune activation, inflammasome signaling, and cellular senescence reinforce one another, contributes to progressive tissue dysfunction, metabolic disease, frailty, and neurodegeneration.

By limiting dysbiosis, barrier dysfunction, and systemic exposure to microbial endotoxins, PHD-consistent dietary patterns may attenuate activation of the TLR4–NF-κB–NLRP3 inflammatory network.

### 4.8. From Microbial Dysbiosis to the Hallmarks of Aging: The Gut Microbiome as a Central Modulator of Biological Aging

The Hallmarks of Aging framework provides a unifying model for understanding the molecular mechanisms underlying biological aging. Increasing evidence indicates that the gut microbiome interacts with several of these interconnected processes, positioning age-related dysbiosis as an active modulator rather than merely a consequence of aging [180].

Loss of microbial diversity, impaired intestinal barrier integrity, altered microbial metabolism, and persistent activation of innate immune pathways reshape the host environment during aging [181]. These changes modify the availability of key microbiome-derived signaling molecules—including short-chain fatty acids, secondary bile acids, indole derivatives, polyphenol metabolites, and endotoxins—which converge on evolutionarily conserved pathways regulating nutrient sensing, mitochondrial homeostasis, cellular maintenance, inflammation, and adaptive stress responses.

Through these interconnected mechanisms, age-related dysbiosis may simultaneously influence autophagy and proteostasis, mitochondrial function, epigenetic regulation, cellular senescence, stem cell function, and intercellular communication [182]. Chronic inflammatory signaling may further reinforce these alterations through self-amplifying feedback loops, allowing dysfunction in one pathway to propagate across multiple hallmarks of aging.

**Figure 2:**
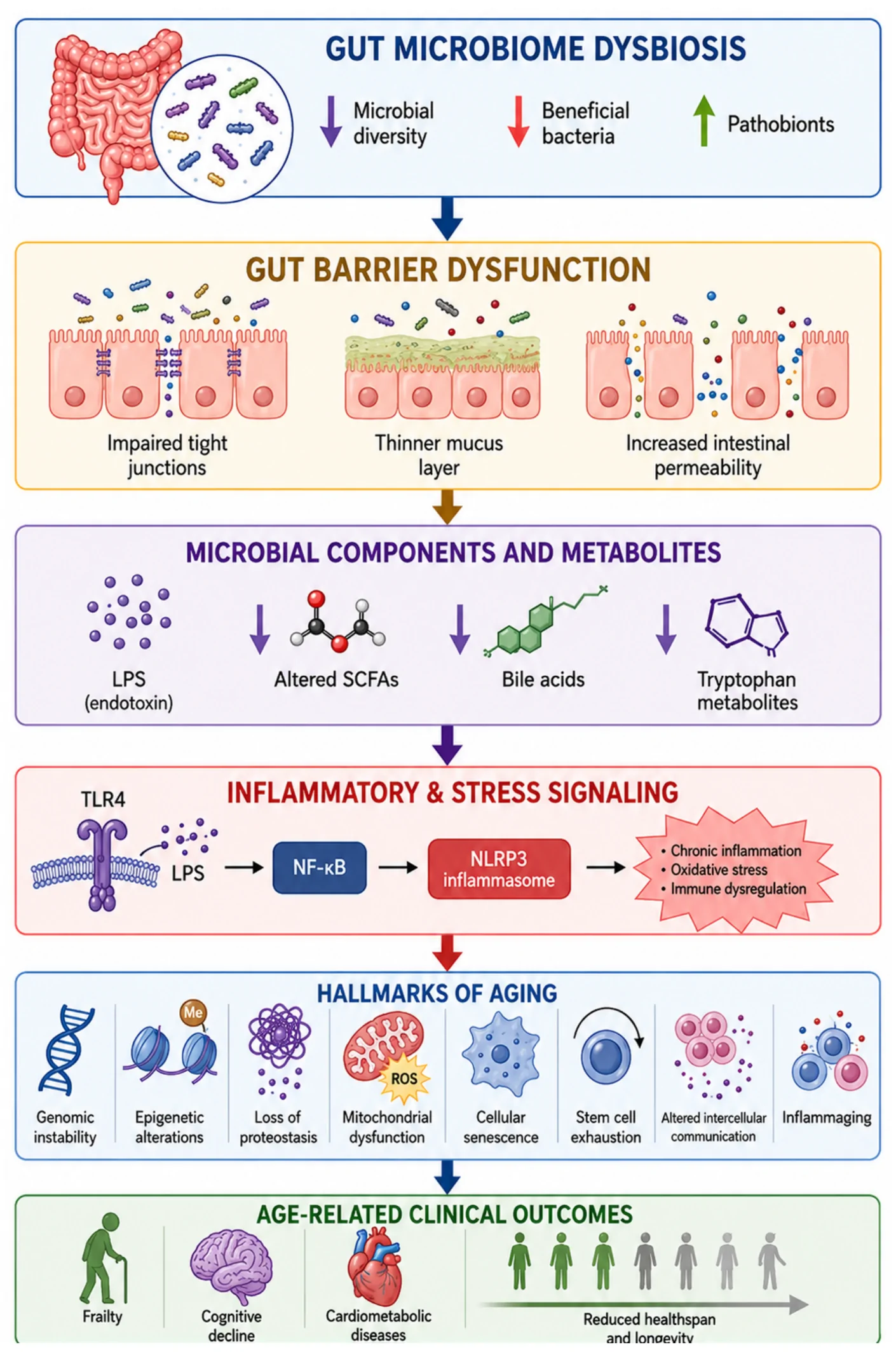
The gut microbiome as a mechanistic link between dysbiosis and the hallmarks of aging. Age-related gut dysbiosis is associated with reduced microbial diversity, loss of beneficial bacteria, increased abundance of pathobionts, impaired intestinal barrier integrity, altered microbial metabolite production, and increased translocation of microbial endotoxins. These changes may activate the TLR4–NF-κB–NLRP3 signaling axis and promote chronic low-grade inflammation, oxidative stress, and immune dysregulation. Gut dysbiosis may thereby contribute to several hallmarks of aging and to frailty, cognitive decline, cardiometabolic disease, reduced healthspan, and shortened lifespan. Arrows between panels indicate the proposed direction of the mechanistic sequence. Downward arrows indicate a decrease, whereas upward arrows indicate an increase. Abbreviations: LPS, lipopolysaccharide; SCFAs, short-chain fatty acids; TLR4, Toll-like receptor 4; NF-κB, nuclear factor kappa B; NLRP3, NLR family pyrin domain-containing 3 inflammasome.

Rather than targeting individual molecular pathways, adherence to the Planetary Health Diet may support microbial ecosystem function by promoting microbial diversity, preserving intestinal barrier integrity, increasing the production of beneficial metabolites, and limiting pro-inflammatory signaling. Section 5 examines the principal signaling networks involved, with their key characteristics and potential modulation by the Planetary Health Diet summarized in Table 2, whereas Section 6 considers their consequences for specific hallmarks of aging (Figure 2).

## 5. Mechanistic Pathways Linking the Planetary Health Diet to Longevity

Recent advances in geroscience have established that biological aging is regulated by interconnected nutrient-sensing and stress-response pathways rather than by chronological time alone [183,184]. These evolutionarily conserved signaling networks integrate environmental inputs, including dietary composition and microbiome-derived metabolites, to coordinate cellular maintenance, metabolic adaptation, and physiological resilience [185].

As discussed in the preceding sections, PHD-consistent dietary patterns may support a gut microbial ecosystem capable of generating a diverse repertoire of bioactive metabolites, including short-chain fatty acids, secondary bile acids, indole derivatives, and polyphenol-derived metabolites [84,186,187]. Rather than acting through independent mechanisms, these microbial signals converge on a limited number of highly conserved molecular pathways that regulate energy metabolism, mitochondrial homeostasis, autophagy, inflammation, and adaptive stress responses [188,189,190].

The following sections examine the evidence supporting potential microbiome-mediated modulation of AMPK, mTOR, IGF-1, SIRT1, FOXO, and autophagy, and discuss how these pathways may contribute to a proposed biological framework linking PHD-consistent dietary patterns with biological aging and healthspan. Direct human evidence for the complete PHD–microbiome–signaling–healthy aging pathway remains limited.

### 5.1. AMP-Activated Protein Kinase: A Central Regulator of Cellular Energy Homeostasis

AMPK is a highly conserved nutrient-sensing kinase that coordinates cellular responses to energy deficiency and functions as a master regulator of metabolic homeostasis [191]. Activated by increases in the AMP/ATP and ADP/ATP ratios, AMPK promotes ATP-generating pathways while suppressing energy-consuming anabolic processes. Beyond its metabolic role, AMPK integrates nutrient availability, mitochondrial function, and cellular stress responses, making it one of the principal regulators of healthy aging [192].

Experimental studies and indirect human evidence from PHD-consistent dietary components suggest that microbiome-derived metabolites may influence AMPK signaling [20,97,105,107,193,194,195,196,197,198,199,200]. Dietary fiber fermentation increases the production of SCFAs, particularly butyrate and propionate, which can activate AMPK directly and through G protein-coupled receptor signaling [201]. Microbiome-derived polyphenol metabolites, including urolithin A and phenolic acids, may further enhance AMPK activity, linking plant-rich dietary patterns with improved metabolic regulation and cellular adaptation [69,202].

Activation of AMPK shifts cellular metabolism toward energy conservation by stimulating fatty acid oxidation, glucose uptake, mitochondrial biogenesis, and autophagy while suppressing lipogenesis and other anabolic pathways [203]. These coordinated adaptations improve mitochondrial efficiency, preserve metabolic flexibility, and enhance resistance to oxidative and metabolic stress, all of which progressively decline during aging [202].

AMPK also occupies a central position within the longevity signaling network through extensive crosstalk with other nutrient-sensing pathways. It suppresses mechanistic target of rapamycin (mTOR) signaling, facilitates SIRT1 activation by increasing cellular NAD^+^ availability, promotes autophagy, and supports mitochondrial quality control [81]. Through these interconnected actions, AMPK integrates microbiome-derived metabolic signals with the molecular pathways that govern cellular maintenance, stress resistance, and healthy aging [204,205].

### 5.2. Mechanistic Target of Rapamycin: Balancing Growth and Longevity

The mechanistic target of rapamycin (mTOR) is a highly conserved nutrient-sensing kinase that coordinates cellular growth with nutrient availability, energy status, and growth factor signaling [206]. Although transient activation of mTORC1 is essential for tissue growth and repair, persistent mTORC1 activation promotes several hallmarks of aging, including impaired autophagy, loss of proteostasis, mitochondrial dysfunction, and the progressive accumulation of cellular damage [207]. Healthy aging depends not on complete inhibition of mTOR, but on maintaining an appropriate balance between anabolic growth and cellular maintenance.

Dietary characteristics consistent with the PHD may influence physiological regulation of mTOR through both nutrient-dependent and microbiome-related mechanisms. Microbial fermentation of dietary fiber increases the production of SCFAs, while microbiome-derived bile acid and polyphenol metabolites further modulate nutrient-sensing pathways [208]. Together, these signals may favor a metabolic environment that supports cellular maintenance over chronic anabolic activation.

Dietary protein composition provides an additional mechanism for mTOR regulation. Compared with Western dietary patterns rich in red and processed meat, the Planetary Health Diet emphasizes legumes, nuts, and other plant-derived protein sources while reducing excessive intake of animal protein [209,210,211]. This dietary pattern moderates exposure to branched-chain amino acids, particularly leucine, thereby supporting physiological mTOR signaling without compromising adequate protein intake [209].

Appropriate regulation of mTOR enhances autophagic flux, facilitates the removal of damaged proteins and dysfunctional organelles, preserves proteostasis, and supports mitochondrial quality control [212]. These coordinated adaptations strengthen cellular stress resistance, reduce chronic inflammation, and delay the accumulation of age-related cellular damage [61,213,214].

### 5.3. Insulin-like Growth Factor-1: Linking Nutrient Sensing to Healthy Longevity

The IGF-1 signaling pathway is a highly conserved regulator of growth, nutrient sensing, and metabolic homeostasis [215]. Although IGF-1 is essential for normal development, tissue repair, and maintenance of skeletal muscle, sustained activation of the insulin/IGF-1 axis during adulthood has been associated with accelerated biological aging, impaired cellular stress resistance, and increased susceptibility to several age-related diseases [216]. Accordingly, healthy aging appears to depend on maintaining physiological regulation of IGF-1 signaling rather than maximizing anabolic activity [217].

The Planetary Health Diet may support this balance through complementary dietary and microbiome-mediated mechanisms [218]. By emphasizing legumes, whole grains, nuts, and other plant-derived protein sources while limiting red and processed meat, the diet provides adequate protein intake without excessive stimulation of anabolic signaling. This dietary pattern has been associated with a more favorable metabolic profile and more balanced regulation of nutrient-sensing pathways, including the insulin/IGF-1–mTOR axis [14,21,97,108,119,197,218,219].

The gut microbiome provides an additional level of regulation. Fermentation of dietary fiber generates SCFAs, which improve insulin sensitivity and metabolic flexibility while reducing chronic hyperinsulinemia. In parallel, microbiome-derived metabolites attenuate chronic low-grade inflammation and support metabolic homeostasis, thereby creating physiological conditions that favor appropriate regulation of the insulin/IGF-1 pathway [220,221].

Appropriate modulation of IGF-1 signaling promotes multiple cellular processes associated with healthy aging. Reduced anabolic signaling enhances FOXO activity, facilitates autophagy, improves DNA repair, strengthens antioxidant defenses, and limits cellular senescence, thereby increasing resilience to metabolic and oxidative stress while preserving tissue homeostasis [222,223].

### 5.4. Sirtuin 1: Connecting the Gut Microbiome to Cellular Longevity Programs

SIRT1 is a NAD^+^-dependent deacetylase that orchestrates cellular adaptation to metabolic and environmental stress by coordinating mitochondrial function, energy metabolism, inflammatory responses, and genome maintenance [224,225]. As one of the principal regulators of healthy aging, SIRT1 integrates nutrient availability with cellular stress responses, thereby preserving metabolic homeostasis and physiological resilience. Declining SIRT1 activity has been associated with mitochondrial dysfunction, chronic inflammation, and progressive functional decline during aging [224].

Bioactive compounds generated from PHD-characteristic dietary components, particularly fiber- and polyphenol-derived microbial products, may modulate SIRT1-related pathways, predominantly based on experimental evidence [65]. Dietary fiber fermentation produces butyrate, whereas microbial transformation of polyphenols generates urolithin A, phenolic acids, and related metabolites linked to SIRT1 activation [226]. Together, these microbial products provide a plausible mechanistic link between dietary signals, gut microbial metabolism, and healthy aging [38].

Activation of SIRT1 promotes metabolic adaptation by enhancing fatty acid oxidation, improving glucose homeostasis, and stimulating mitochondrial quality control through deacetylation of PGC-1α [227]. These coordinated adaptations preserve oxidative phosphorylation, maintain ATP production, reduce oxidative stress, and support mitochondrial integrity, all of which progressively decline with advancing age [227].

Beyond its metabolic functions, SIRT1 exerts broad cytoprotective effects by suppressing NF-κB-dependent inflammatory signaling and attenuating the senescence-associated secretory phenotype (SASP), thereby limiting chronic low-grade inflammation and preserving tissue homeostasis [228,229].

SIRT1 functions within an integrated longevity signaling network characterized by extensive crosstalk with AMPK, mTOR, FOXO, and autophagy. Through these coordinated interactions, microbiome-derived metabolites help couple cellular energy status with mitochondrial maintenance, proteostasis, and stress resistance, thereby reinforcing adaptive responses that preserve cellular integrity throughout aging.

### 5.5. Forkhead Box O Transcription Factors: Genetic Programs of Cellular Stress Resistance and Healthy Longevity

FOXO transcription factors are key downstream effectors of conserved nutrient-sensing pathways that coordinate genetic programs involved in cellular maintenance, stress adaptation, and healthy aging [230]. Acting primarily downstream of the insulin/IGF-1 signaling axis, FOXO proteins regulate the expression of genes controlling antioxidant defense, DNA repair, autophagy, apoptosis, and proteostasis, thereby enhancing cellular resilience under metabolic and oxidative stress [230,231]. Sustained FOXO activity is widely regarded as a hallmark of healthy longevity.

FOXO activity is tightly regulated by nutrient availability and cellular energy status. Under nutrient-rich conditions, insulin and IGF-1 activate protein kinase B (Akt), leading to FOXO phosphorylation and its exclusion from the nucleus, thereby suppressing transcription of stress-response genes [232]. Conversely, reduced anabolic signaling together with activation of AMPK and SIRT1 promotes FOXO nuclear localization and transcriptional activation. Microbial metabolites derived from PHD-characteristic dietary components may influence this adaptive stress-response network, although evidence for PHD-specific regulation of FOXO in humans remains indirect [193,233]. SCFAs, indole derivatives, and polyphenol-derived metabolites may modulate the AMPK–SIRT1–FOXO signaling axis [232], thereby enhancing antioxidant defenses, including superoxide dismutase 2 and catalase, and improving resistance to oxidative stress [234]. These adaptive responses may be particularly relevant during aging, when oxidative stress contributes to genomic instability, mitochondrial impairment, and loss of cellular function [232,235].

Beyond antioxidant defense, FOXO coordinates multiple cellular maintenance programs by regulating DNA repair, autophagy, cell-cycle control, and proteostasis [230]. Through these integrated actions, FOXO promotes the removal of damaged proteins and dysfunctional organelles, preserves mitochondrial quality, and counteracts several hallmarks of aging, including proteostatic decline, cellular senescence, and impaired stress adaptation [236,237].

### 5.6. Autophagy: A Central Mechanism of Cellular Quality Control and Renewal

Autophagy is an evolutionarily conserved intracellular quality-control system responsible for the degradation and recycling of damaged proteins, dysfunctional organelles, and other cellular components [238]. By preserving proteostasis, maintaining organelle integrity, and facilitating adaptation to metabolic stress, autophagy is essential for cellular homeostasis throughout life. Age-related decline in autophagic activity leads to the progressive accumulation of cellular damage, mitochondrial dysfunction, oxidative stress, and senescent cells, thereby contributing to functional deterioration across multiple tissues [238].

Impaired autophagy is now recognized as a fundamental hallmark of aging and a major contributor to numerous chronic disorders, including neurodegenerative diseases, cardiometabolic disorders, sarcopenia, and immune dysfunction [61,185,207,239,240].

Several microbial metabolites derived from fiber- and polyphenol-rich foods can modulate pathways involved in autophagy, providing an indirect mechanistic basis for potential effects of PHD-consistent dietary patterns on cellular quality control. Dietary fiber fermentation increases SCFA production, particularly butyrate, whereas microbial biotransformation of polyphenols generates metabolites such as urolithin A, a potent inducer of mitophagy that promotes the selective removal of dysfunctional mitochondria [61,83,241]. Together, these metabolites may support cellular maintenance and renewal.

Autophagy is regulated through extensive crosstalk among multiple longevity-associated signaling pathways. Activation of AMPK, physiological modulation of mTOR, and the coordinated actions of SIRT1 and FOXO promote autophagic flux, proteostasis, and mitochondrial quality control [83,241,242]. These pathways converge on autophagy as a common downstream mechanism that preserves cellular integrity under metabolic and oxidative stress.

Mitophagy represents a specialized component of this quality-control system that selectively eliminates damaged mitochondria, thereby maintaining ATP production, limiting reactive oxygen species generation, and preserving metabolic flexibility [243]. By preventing mitochondrial dysfunction and the accumulation of oxidative damage, mitophagy counteracts several interconnected hallmarks of aging, including cellular senescence and loss of physiological resilience [243,244].

### 5.7. Mitochondrial Function: The Final Effector of Cellular Energy Homeostasis and Healthy Longevity

Mitochondria are the central regulators of cellular energy metabolism, extending far beyond ATP production to coordinate redox homeostasis, calcium signaling, apoptosis, innate immunity, and metabolic adaptation [245]. As multifunctional signaling organelles, they integrate nutrient availability with cellular stress responses, making mitochondrial integrity a critical determinant of physiological resilience throughout aging. Accordingly, mitochondrial dysfunction is recognized as a fundamental hallmark of aging and a major contributor to functional decline across multiple organ systems [246].

Advancing age is accompanied by progressive deterioration of mitochondrial function, characterized by impaired oxidative phosphorylation, reduced ATP generation, excessive production of reactive oxygen species (ROS), and the accumulation of dysfunctional mitochondria [247]. These alterations disrupt cellular bioenergetics, amplify chronic inflammation, and promote cellular senescence, thereby contributing to the development of cardiometabolic, neurodegenerative, and musculoskeletal disorders [247].

Evidence from studies of dietary fiber, polyphenols, and selected microbiome-derived metabolites suggests that mechanisms relevant to PHD-consistent dietary patterns may converge on mitochondrial function, although direct PHD-specific evidence remains limited [24,59,248]. SCFAs, particularly butyrate, support oxidative metabolism and mitochondrial adaptation, whereas microbial transformation of polyphenols generates compounds such as urolithin A, a well-characterized inducer of mitophagy that facilitates the selective removal of dysfunctional mitochondria [173,174,175,248]. Together, these effects may support mitochondrial quality control and metabolic flexibility.

Beyond supporting mitochondrial turnover, microbiome-derived metabolites improve respiratory chain efficiency, reduce oxidative stress, and preserve mitochondrial DNA integrity, thereby sustaining cellular energy production in metabolically active tissues such as skeletal muscle, the heart, and the brain [248].

Mitochondria also occupy a central position at the interface between metabolism and innate immunity. Dysfunctional mitochondria release mitochondrial DNA and other damage-associated molecular patterns that activate the NLRP3 inflammasome, thereby reinforcing chronic low-grade inflammation [249]. By preserving mitochondrial quality control, the Planetary Health Diet may attenuate inflammasome activation while simultaneously maintaining cellular bioenergetics, illustrating the close relationship between metabolic resilience and immune homeostasis [193].

Rather than representing an isolated biological process, mitochondrial function integrates the coordinated actions of the longevity-associated pathways discussed throughout this review. Regulation of AMPK, mTOR, IGF-1, SIRT1, FOXO, and autophagy ultimately converges on the preservation of mitochondrial quality, energy homeostasis, and cellular resilience.

### 5.8. Oxidative Stress: Microbiome-Mediated Regulation of Redox Homeostasis

Oxidative stress arises when the generation of ROS exceeds the capacity of endogenous antioxidant defense systems, resulting in cumulative oxidative damage to lipids, proteins, and nucleic acids [250]. Although ROS play essential roles in physiological signaling and cellular adaptation, persistent redox imbalance contributes to genomic instability, mitochondrial dysfunction, chronic inflammation, and cellular senescence. Oxidative stress is increasingly recognized as a common mechanistic feature linking multiple hallmarks of aging.

Age-related alterations in the gut microbiome further amplify oxidative stress. Loss of SCFA-producing bacteria, impaired intestinal barrier integrity, endotoxin translocation, and chronic low-grade inflammation promote mitochondrial ROS production while weakening endogenous antioxidant defenses [251]. Together, these processes establish a self-reinforcing cycle in which oxidative stress and inflammation progressively exacerbate one another during aging.

PHD-consistent dietary characteristics may contribute to redox homeostasis through effects on gut microbial metabolism and the production of bioactive compounds. SCFAs, particularly butyrate, and polyphenol-derived metabolites modulate stress-response pathways that enhance endogenous antioxidant capacity and mitochondrial function [65,252]. Rather than acting primarily as direct free radical scavengers, these compounds promote adaptive cellular responses that increase resistance to oxidative stress. Microbial transformation of polyphenols also generates urolithin A and low-molecular-weight phenolic acids, which activate cytoprotective pathways and reinforce cellular resilience through hormetic signaling [68,69,253].

In this context, the antioxidant effects of the Planetary Health Diet appear to arise not simply from its high content of antioxidant compounds, but from its capacity to remodel gut microbial metabolism and strengthen endogenous defense systems.

### 5.9. Epigenetic Regulation: The Gut Microbiome as a Modulator of Longevity-Associated Gene Expression

Epigenetic regulation provides a mechanistic framework through which environmental exposures, including diet and the gut microbiome, exert long-term effects on biological aging without altering the underlying DNA sequence [254,255]. Age-related changes in DNA methylation, histone modifications, and non-coding RNA expression progressively remodel gene networks involved in inflammation, metabolism, stress adaptation, cellular senescence, and tissue repair. Unlike genetic variation, these epigenetic modifications remain dynamically responsive to environmental influences, making them attractive targets for dietary strategies that promote healthy aging.

The Planetary Health Diet may influence epigenetic regulation by reshaping the metabolic activity of the gut microbiome. Among microbiome-derived metabolites, butyrate is one of the best-characterized endogenous epigenetic modulators [256]. As a natural histone deacetylase inhibitor, butyrate increases histone acetylation and chromatin accessibility, thereby facilitating the transcription of genes involved in antioxidant defense, immune regulation, and cellular stress adaptation [32,257]. This mechanism provides a plausible molecular pathway linking microbial fermentation of dietary fiber with the regulation of host gene expression.

The gut microbiome may further influence epigenetic programming through one-carbon metabolism. By contributing to the availability of folate and other methyl-donor metabolites, microbial metabolism affects DNA methylation processes and may influence genome-wide methylation patterns associated with aging [258]. Consistent with these observations, adherence to healthy plant-based dietary patterns has been associated with slower epigenetic aging, as estimated by DNA methylation-based biological aging clocks [259].

Non-coding RNAs, particularly microRNAs, provide an additional level of epigenetic regulation by fine-tuning post-transcriptional gene expression. Experimental and emerging clinical evidence suggests that gut microbial composition and metabolite production influence microRNAs involved in inflammatory signaling, mitochondrial function, oxidative stress responses, and autophagy, providing another pathway through which diet may affect cellular aging [260,261].

Epigenetic mechanisms also interact with multiple longevity-associated signaling pathways. AMPK, SIRT1, FOXO, autophagy, and mitochondrial metabolism interact with epigenetic regulators to coordinate cellular adaptation, stress resistance, and metabolic homeostasis. Through these interactions, microbiome-derived metabolites may contribute to transcriptional and epigenetic responses to dietary exposures.

## 6. Gut Microbiome and the Hallmarks of Aging

The Hallmarks of Aging framework provides a unifying biological model for understanding how molecular and cellular processes collectively drive the progressive decline in physiological function during aging [262]. These hallmarks are interconnected through extensive biological crosstalk, forming an integrated network that shapes biological aging and susceptibility to age-related diseases. Recent refinements of this framework have further emphasized chronic inflammation, altered intercellular communication, and gut microbial dysbiosis as important contributors to the aging process [232].

The gut microbiome is increasingly recognized as an upstream modulator of multiple hallmarks of aging rather than as a regulator of any single biological pathway [24]. Age-related dysbiosis alters microbial metabolite production, compromises intestinal barrier integrity, and promotes chronic low-grade inflammation, thereby potentially influencing genomic stability, epigenetic regulation, mitochondrial function, proteostasis, cellular senescence, stem cell function, and immune homeostasis [171,263,264]. Conversely, microbial ecosystems associated with PHD-consistent dietary patterns may generate bioactive metabolites that support cellular maintenance, adaptive stress responses, and physiological resilience. The following sections examine how these microbiome-mediated processes may interact with individual hallmarks of aging, while distinguishing experimental evidence from findings demonstrated in human populations.

### 6.1. Genomic Instability

Genomic instability is a primary hallmark of aging and reflects the progressive accumulation of DNA damage, impaired DNA repair, and loss of chromosomal integrity [183,232,265]. Oxidative stress, mitochondrial dysfunction, and chronic low-grade inflammation accelerate genomic injury, thereby promoting cellular senescence, tissue dysfunction, and susceptibility to age-related diseases.

The gut microbiome may influence genomic stability primarily through its effects on oxidative stress and inflammatory signaling. Microbiome-derived SCFAs, particularly butyrate, may support DNA repair and reduce oxidative DNA damage, whereas gut dysbiosis, metabolic endotoxemia, and sustained pro-inflammatory signaling may increase genomic injury [266].

### 6.2. Epigenetic Alterations

Epigenetic alterations are a defining hallmark of aging and involve progressive changes in gene regulation without alteration of the underlying DNA sequence [189,254,267]. Age-related changes in DNA methylation, histone modifications, and non-coding RNA expression contribute to dysregulated inflammation, metabolic dysfunction, impaired stress adaptation, and accelerated biological aging.

The gut microbiome may influence epigenetic homeostasis through the production of bioactive metabolites. SCFAs, particularly butyrate, modulate chromatin accessibility through histone deacetylase inhibition, while microbial metabolism may also affect DNA methylation and non-coding RNA pathways involved in inflammation, mitochondrial function, and cellular maintenance [268].

### 6.3. Loss of Proteostasis

Loss of proteostasis is a fundamental hallmark of aging and reflects the progressive deterioration of cellular systems responsible for protein synthesis, folding, quality control, and degradation [232]. As these systems decline, damaged and misfolded proteins accumulate, contributing to cellular dysfunction, neurodegenerative diseases, sarcopenia, and metabolic disorders [269,270].

The gut microbiome may influence proteostasis through its effects on inflammation, oxidative stress, and cellular quality-control pathways [271]. Age-related dysbiosis promotes a proteotoxic environment characterized by chronic low-grade inflammation and oxidative damage, whereas microbiome-derived metabolites may support adaptive stress responses and limit the accumulation of damaged proteins [272,273].

Proteostasis is closely linked to autophagy and mitophagy, which remove damaged proteins and dysfunctional organelles [232]. By supporting these quality-control processes, microbiome-derived metabolites may help preserve protein turnover and cellular function during aging.

### 6.4. Mitochondrial Dysfunction

Mitochondrial dysfunction is a central hallmark of aging and is characterized by impaired oxidative phosphorylation, reduced ATP production, excessive ROS generation, and progressive loss of mitochondrial quality control [274,275]. These alterations compromise cellular bioenergetics, disrupt metabolic homeostasis, and contribute to chronic inflammation, cellular senescence, and age-related disease.

The gut microbiome may influence mitochondrial homeostasis through the production of bioactive metabolites, including SCFAs, secondary bile acids, indole derivatives, and polyphenol-derived metabolites [24,248]. These metabolites may support oxidative metabolism, mitochondrial integrity, and adaptive stress responses.

By promoting microbial functions associated with the production of these metabolites, PHD-consistent dietary patterns may help preserve mitochondrial quality control, limit oxidative stress, and maintain cellular energy homeostasis and metabolic flexibility during aging [276,277].

Mitochondrial dysfunction may also amplify inflammaging through the release of mitochondrial DNA and other damage-associated molecular patterns that activate innate immune pathways, including the NLRP3 inflammasome [83,232,274]. Preservation of mitochondrial integrity may therefore attenuate inflammatory signaling while supporting metabolic and immune homeostasis.

### 6.5. Cellular Senescence

Cellular senescence is a fundamental hallmark of aging characterized by irreversible cell-cycle arrest accompanied by sustained metabolic activity [278]. Although senescence has important physiological roles in tumor suppression, tissue repair, and wound healing, the progressive accumulation of senescent cells contributes to tissue dysfunction and chronic disease [279].

Senescent cells develop a senescence-associated secretory phenotype (SASP), characterized by the release of pro-inflammatory cytokines, chemokines, growth factors, and proteases [280]. Persistent SASP signaling disrupts tissue homeostasis, promotes chronic low-grade inflammation, and may propagate senescence to neighboring cells [281].

Age-related gut dysbiosis may favor cellular senescence by promoting metabolic endotoxemia, oxidative stress, and sustained inflammatory signaling [6,282]. Reduced production of beneficial microbial metabolites may further impair cellular maintenance and adaptive stress responses [8].

Conversely, microbiome-derived SCFAs and polyphenol metabolites associated with PHD-consistent dietary patterns may attenuate inflammatory signaling, support mitochondrial homeostasis, and limit oxidative stress [179,283]. These effects may reduce SASP-associated inflammation and help preserve tissue homeostasis.

### 6.6. Stem Cell Exhaustion

Stem cell exhaustion is a defining hallmark of aging and is characterized by the progressive decline in the abundance, regenerative capacity, and functional competence of tissue-specific stem cells. With advancing age, hematopoietic, intestinal, skeletal muscle, neural, and epidermal stem cell populations gradually lose their capacity for self-renewal, contributing to impaired tissue repair, delayed regeneration, and progressive functional decline [183,232,284,285].

The gut microbiome may influence stem cell homeostasis through its effects on the local and systemic stem cell niche [286,287]. Age-related dysbiosis promotes chronic low-grade inflammation, metabolic disturbance, oxidative stress, and immune dysregulation, thereby creating a microenvironment that may impair stem cell function. Persistent inflammatory signaling can disrupt self-renewal and tissue regeneration, particularly in hematopoietic and intestinal compartments [24,166].

Microbiome-derived metabolites may also contribute to the preservation of stem cell function. SCFAs support intestinal barrier integrity and epithelial energy metabolism, whereas indole derivatives and secondary bile acids may influence signaling pathways involved in stem cell maintenance, differentiation, and tissue homeostasis, including AhR- and FXR-dependent pathways [288].

By promoting a fiber-rich dietary pattern and microbial functions associated with the production of these metabolites, PHD-consistent dietary patterns may help maintain a less inflammatory and metabolically favorable stem cell niche [289]. These microbiome-mediated effects may support regenerative capacity, tissue repair, and physiological resilience during aging [38].

### 6.7. Altered Intercellular Communication and Chronic Inflammation

Altered intercellular communication is a defining hallmark of aging and reflects the progressive disruption of coordinated signaling among the immune, endocrine, nervous, and metabolic systems [232]. With advancing age, deterioration of these interconnected networks contributes to chronic low-grade inflammation, immunosenescence, and reduced physiological resilience [290].

The gut microbiome may influence this communication network through the production of bioactive metabolites, including SCFAs, secondary bile acids, and tryptophan-derived indoles, which participate in bidirectional signaling along the gut–immune–brain axis and modulate immune, metabolic, endocrine, and neuroendocrine function [291,292].

Age-related dysbiosis may disrupt these signaling pathways through loss of butyrate-producing bacteria, impaired intestinal barrier integrity, and increased translocation of microbial products. These changes promote persistent inflammatory signaling and increased production of cytokines such as IL-6, TNF-α, and IL-1β [293,294]. Systemic propagation of these signals may contribute to endothelial dysfunction, cardiometabolic disease, neurodegeneration, frailty, and reduced physiological resilience [294].

Conversely, microbiome-derived metabolites associated with PHD-consistent dietary patterns may support immune tolerance, intestinal barrier integrity, and more balanced immunometabolic signaling [21,289]. SCFAs may promote regulatory T-cell differentiation, while polyphenol-derived metabolites, secondary bile acids, and indole derivatives may modulate signaling through receptors such as AhR, FXR, and TGR5.

### 6.8. Gut Dysbiosis as an Emerging Hallmark of Aging

In classical models of aging, alterations in the gut microbiome were largely regarded as secondary consequences of age-related physiological decline [263]. Growing evidence from geroscience, however, suggests that gut dysbiosis may also contribute to biological aging by influencing multiple interconnected molecular and cellular processes [204]. This perspective positions the gut microbiome as a dynamic modulator of systemic aging rather than merely a passive marker of chronological or biological decline.

Age-associated dysbiosis is characterized by reduced microbial diversity, depletion of beneficial metabolite-producing taxa, impaired intestinal barrier integrity, and altered microbial metabolism [295]. These changes may promote chronic low-grade inflammation, metabolic dysfunction, oxidative stress, and impaired cellular maintenance, thereby affecting genomic stability, epigenetic regulation, proteostasis, mitochondrial function, cellular senescence, stem cell homeostasis, and intercellular communication [296]. Rather than acting through a single pathway, dysbiosis may influence several hallmarks of aging through convergent effects on microbial metabolite production, barrier function, and inflammatory signaling.

A distinctive feature of the gut microbiome is its substantial plasticity across the life course and its responsiveness to environmental exposures, particularly diet. PHD-consistent dietary patterns may support a more functionally resilient microbial ecosystem, increase the production of beneficial microbial metabolites, and limit pathways associated with pro-inflammatory microbial products [218]. Through these microbiome-mediated effects, dietary exposures may influence conserved longevity-associated signaling pathways and several hallmarks of aging simultaneously.

Current evidence supports considering gut dysbiosis as an emerging component of the biological aging framework and as a plausible mechanistic interface between sustainable nutrition and healthy aging. However, longitudinal and interventional human studies are still needed to determine whether dysbiosis fulfills the criteria of an independent hallmark and to establish the direction and causality of these relationships. Modulation of the gut microbiome through PHD-consistent dietary patterns may nevertheless represent a potentially useful strategy for preserving physiological resilience and supporting healthy aging across the life course.

## 7. Organ-Specific Effects of the Planetary Health Diet–Gut Microbiome Axis

The gut microbiome is increasingly recognized as a systemic modulator of human physiology rather than solely a determinant of intestinal health. The biological effects of the Planetary Health Diet therefore extend far beyond the gastrointestinal tract, reflecting coordinated communication along multiple gut–organ axes [162,297].

Microbiome-derived metabolites—including SCFAs, tryptophan-derived indoles, secondary bile acids, and polyphenol-derived metabolites—serve as key mediators of this interorgan communication [162].

Although each organ responds through distinct biological pathways, many of the beneficial effects of the Planetary Health Diet converge on common mechanisms, including attenuation of chronic low-grade inflammation, preservation of barrier integrity, optimization of cellular bioenergetics, and maintenance of tissue resilience [19,48,108,298,299,300].

Table 3 summarizes the principal gut–organ axes through which the Planetary Health Diet-associated gut microbiome influences the brain, cardiovascular system, skeletal muscle, liver, immune system, and bone, highlighting both shared and tissue-specific mechanisms that contribute to healthspan and healthy aging.

**Figure 3:**
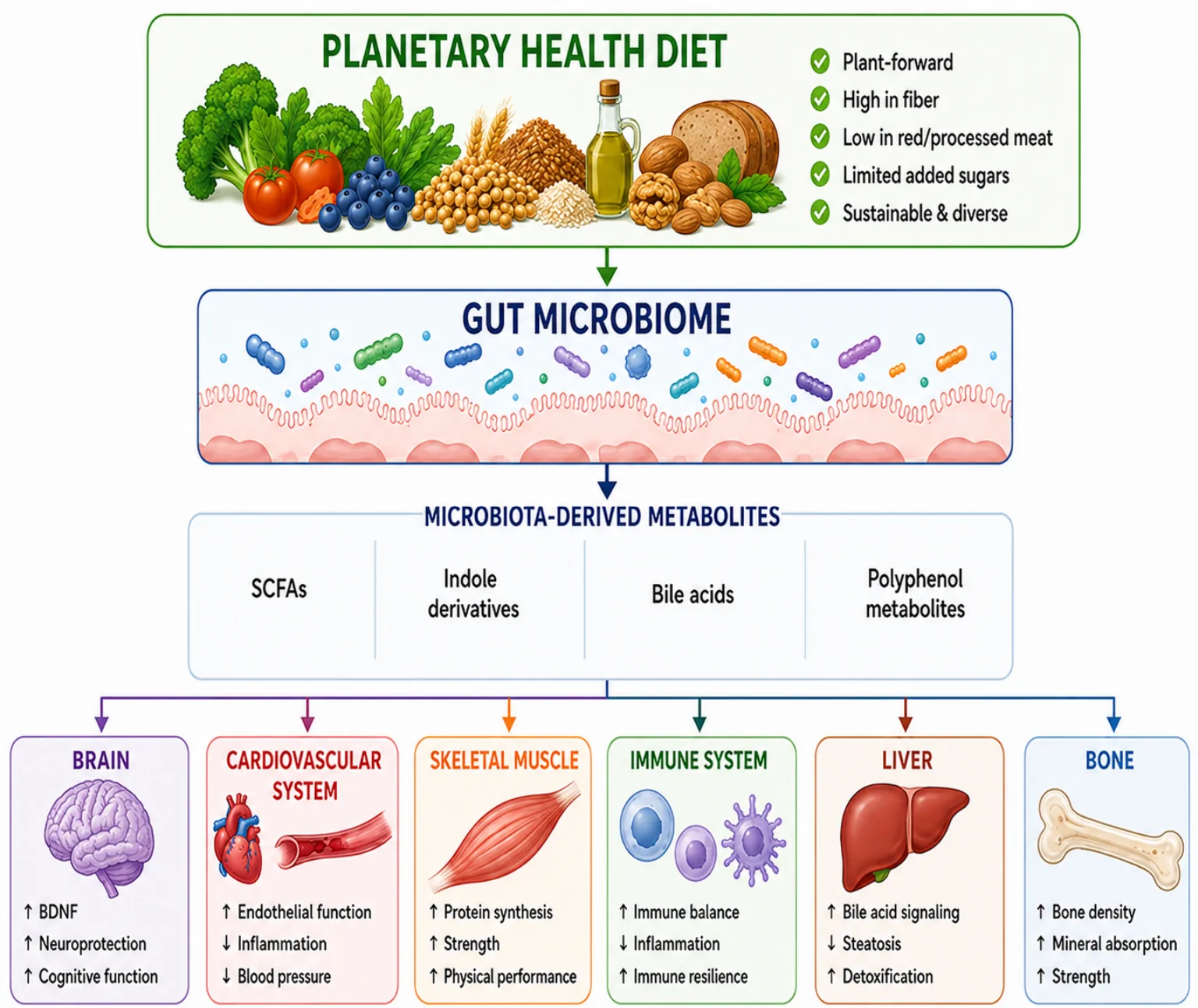
Organ-specific effects mediated by the Planetary Health Diet–gut microbiome axis. The Planetary Health Diet may support a diverse and metabolically active gut microbiome and promote the production of microbiota-derived metabolites involved in gut–organ communication. These metabolites may modulate immune and metabolic homeostasis, mitochondrial function, and tissue-specific physiological processes in the brain, cardiovascular system, skeletal muscle, immune system, liver, and bone. Arrows indicate the proposed direction of effects; upward arrows indicate an increase or enhancement, whereas downward arrows indicate a decrease or reduction. Abbreviations: SCFAs, short-chain fatty acids; BDNF, brain-derived neurotrophic factor.

The molecular mechanisms described above converge on organ-specific effects mediated by gut microbiome-derived signaling, thereby supporting physiological resilience and healthy aging across multiple organ systems (Figure 3).

## 8. Clinical Evidence Supporting the Planetary Health Diet–Healthy Aging Axis

The preceding sections have outlined the molecular and microbiome-mediated mechanisms through which the Planetary Health Diet may promote healthy aging. Increasingly, findings from human studies are consistent with these biological mechanisms, providing translational support for the proposed links between sustainable dietary patterns, gut microbial function, and healthy aging.

Although relatively few clinical studies have directly characterized the gut microbiome, accumulating evidence indicates that dietary patterns aligned with the principles of the Planetary Health Diet are associated with favorable aging-related outcomes. Across prospective cohort studies, randomized controlled trials, and systematic reviews, greater adherence to these dietary patterns has been associated with lower all-cause and cardiovascular mortality, reduced cardiometabolic disease risk, improved cognitive function, greater functional capacity, lower frailty risk, and increased healthy life expectancy. These outcomes represent different levels of evidence and should not be considered equivalent measures of healthy aging. Functional capacity, intrinsic capacity, frailty, healthspan, and longevity are considered direct aging-related outcomes, whereas disease incidence and cardiometabolic, inflammatory, or molecular measures are interpreted as supportive intermediate outcomes.

The strongest epidemiological evidence derives from large prospective cohort studies, including NHANES, UK Biobank, the Nurses’ Health Study, Nurses’ Health Study II, the Health Professionals Follow-up Study, EPIC-Oxford, SUN, the Million Veteran Program, and Seniors-ENRICA, together encompassing several million participants [18,194,198,324,325,326,327,328,329,330,331,332].

Complementary evidence is provided by landmark randomized controlled trials, particularly PREDIMED and CORDIOPREV. Because Mediterranean dietary patterns substantially overlap with the Planetary Health Diet in their emphasis on plant foods, dietary fiber, unsaturated fats, and polyphenol-rich foods, these trials provide supportive but indirect evidence and should not be interpreted as direct evaluations of the PHD. Nevertheless, they demonstrate that Mediterranean dietary patterns reduce major cardiovascular events, lower the incidence of type 2 diabetes, and preserve cognitive function [333,334,335,336,337]. In addition, umbrella reviews and meta-analyses consistently indicate that Mediterranean and predominantly plant-based dietary patterns confer broad benefits across multiple domains of healthy aging, including cardiovascular health, metabolic function, neurodegeneration, frailty, and longevity [338,339].

Although direct microbiome-based intervention studies remain limited, existing epidemiological and clinical findings support the hypothesis that modulation of the gut microbiome represents one plausible biological pathway through which the Planetary Health Diet contributes to healthy aging. Table 4 summarizes representative prospective cohort studies, randomized controlled trials, systematic reviews, and meta-analyses evaluating the associations between the Planetary Health Diet—or closely related dietary patterns—and major healthy aging outcomes.

Although most available data originate from observational cohort studies and only a limited number of investigations have directly characterized the gut microbiome, the overall consistency across diverse populations, study designs, and clinical outcomes supports the biological plausibility of the microbiome-mediated mechanisms discussed throughout this review. Future randomized controlled trials integrating dietary assessment with longitudinal multi-omics profiling will be essential to clarify the causal microbiome pathways underlying healthy aging.

## 9. Environmental Sustainability and Healthy Longevity

Healthy aging can no longer be regarded solely as a biomedical objective. Population aging, the growing burden of chronic non-communicable diseases, climate change, biodiversity loss, and unsustainable food systems are increasingly recognized as interconnected global challenges requiring coordinated responses [2]. This perspective is consistent with the One Health and Planetary Health frameworks, which emphasize the interdependence of human, animal, and environmental health.

Current food systems contribute substantially to greenhouse gas emissions, freshwater use, land-use change, biodiversity loss, and the global burden of chronic disease. At the same time, dietary patterns characterized by high intakes of red and processed meat and ultra-processed foods are associated with increased risks of obesity, type 2 diabetes, cardiovascular disease, cancer, and other age-related disorders [340]. Improving dietary quality therefore offers an opportunity to address environmental pressures and population health within a shared preventive framework.

Within this context, the Planetary Health Diet aligns nutritional recommendations with environmental sustainability by emphasizing plant-based foods and limiting environmentally intensive animal-source products [195]. These dietary characteristics may also support a more diverse and metabolically active gut microbiome, providing a plausible biological connection between sustainable nutrition and healthy aging [341].

The One Health and Planetary Health frameworks further highlight that sustainable food systems generate benefits beyond individual health, including preservation of biodiversity, protection of soil and water resources, improved food security, and reduced environmental pressures [342,343,344]. This perspective is particularly relevant for rapidly aging societies, where extending lifespan without preserving functional capacity places increasing pressure on healthcare and social support systems. Sustainable aging therefore requires strategies that maintain healthspan, intrinsic capacity, and independence while reducing the environmental burden of food production [32].

By aligning plant-rich dietary patterns with lower-resource food production, the PHD provides a population-level framework that may support functional aging while reducing the environmental pressures associated with current food systems [32,342,343,344,345,346,347].

## 10. Clinical and Public Health Implications for Healthy Longevity

### 10.1. Clinical Implications

The PHD has considerable potential not only for the primary prevention of chronic diseases but also as part of secondary prevention strategies and lifestyle interventions that support healthy aging. Growing evidence suggests that nutritional interventions can simultaneously influence multiple biological processes involved in aging, thereby delaying functional decline, improving physiological resilience, and reducing the burden of multimorbidity [14].

In older adults, however, implementation of the PHD should be individualized according to nutritional status, functional capacity, and existing comorbidities. Maintaining adequate protein intake is particularly important for preserving skeletal muscle mass and strength, with emphasis on high-quality plant protein sources such as legumes, soy products, nuts, and seeds, complemented by moderate amounts of animal-derived foods when clinically appropriate. Long-term adherence to predominantly plant-based dietary patterns may also require monitoring of vitamin B12, vitamin D, calcium, iron, iodine, and long-chain omega-3 fatty acid status in selected individuals to ensure nutritional adequacy. Importantly, the PHD is not a rigid vegetarian diet but a flexible dietary framework that can be adapted to age, clinical status, cultural traditions, regional food availability, and individual nutritional requirements [218].

### 10.2. Public Health Implications

At the population level, greater adherence to PHD-consistent dietary patterns may reduce the incidence of cardiometabolic diseases, frailty, disability, and premature mortality, with potential downstream reductions in healthcare utilization and costs [340]. Realizing these benefits will require affordable access to nutrient-dense plant foods, culturally adaptable dietary guidance, and coordinated implementation across healthcare, education, and food-policy settings.

Public health strategies should also address socioeconomic inequalities that influence food affordability, dietary quality, and access to preventive services. Particular attention is needed for older adults, socioeconomically disadvantaged populations, and individuals living in institutional or food-insecure settings. Population-level implementation should therefore be accompanied by monitoring of dietary adequacy, health outcomes, equity, acceptability, and environmental impact to ensure that the transition toward PHD-consistent dietary patterns produces inclusive and sustainable benefits.

### 10.3. Alignment with the WHO Healthy Ageing Framework

The principles of the Planetary Health Diet closely align with the objectives of the World Health Organization Decade of Healthy Ageing (2021–2030), which emphasizes maintaining functional ability, preserving intrinsic capacity, preventing disability, and promoting healthy aging throughout the life course [348]. Through potential effects on metabolic, immune, and microbiome-related pathways, the PHD may contribute to maintaining physical, cognitive, and metabolic function in later life [349].

As evidence continues to accumulate, the PHD may provide a practical nutritional framework for translating advances in aging biology into preventive strategies aimed at preserving physiological resilience and functional independence among older adults [350].

### 10.4. Policy Implications

Successful implementation of the Planetary Health Diet requires supportive public policies that extend beyond individual dietary choices. National dietary guidelines, food procurement policies, agricultural strategies, and healthcare systems should increasingly integrate sustainability with health promotion. Public institutions—including schools, hospitals, nursing homes, and long-term care facilities—provide important opportunities to implement dietary practices that simultaneously support healthy aging and environmental sustainability [17].

Policy initiatives should improve access to affordable fruits, vegetables, legumes, whole grains, and other nutrient-dense plant foods, encourage sustainable food production, reduce food waste, and strengthen nutrition education throughout the life course. Integrating sustainability into national dietary guidelines may facilitate the transition toward food systems that improve population health, reduce healthcare costs, and mitigate the environmental impacts of food production [351].

### 10.5. Future Implementation

Future implementation of the Planetary Health Diet will require coordinated action across clinical practice, public health, food policy, agriculture, and environmental sustainability. Multidisciplinary collaboration among clinicians, nutrition scientists, public health professionals, policymakers, agricultural experts, and environmental scientists will be essential to translate PHD principles into culturally acceptable, nutritionally adequate, affordable, and scalable interventions. Implementation strategies should also be adapted to population needs, healthcare settings, regional food systems, and socioeconomic conditions. Monitoring of adherence, nutritional adequacy, equity, health outcomes, and environmental impact will be necessary to ensure that PHD-consistent dietary transitions generate sustainable and inclusive benefits across the life course [352].

## 11. Knowledge Gaps and Future Perspectives

Despite rapidly expanding evidence supporting the potential benefits of the Planetary Health Diet for the gut microbiome and healthy aging, important knowledge gaps remain. Much of the current evidence is derived from observational cohort studies, limiting causal inference regarding the relationships among dietary adherence, microbiome remodeling, microbial metabolism, and long-term health outcomes. Future research should therefore prioritize large prospective cohort studies and well-designed randomized controlled trials integrating clinical, nutritional, microbiome, multi-omics, and molecular data.

A major challenge is the substantial interindividual variability in gut microbiome composition and function. Individuals consuming similar dietary patterns often exhibit markedly different microbial responses because of differences in genetics, age, medication use, lifestyle, environmental exposures, and baseline microbial ecology. This variability highlights the need for precision nutrition approaches tailored to individual biological characteristics rather than universal dietary recommendations.

Population-level variability represents an additional challenge. Although PHD adherence indices have been developed and applied across several populations, their validation and epidemiological use remain geographically uneven, with limited representation of many non-Western populations [16]. This is particularly relevant to microbiome-based interpretations, because gut microbial composition and function vary substantially with geography, ethnicity, habitual diet, lifestyle, and environmental exposures [353]. Consequently, similar PHD adherence scores may not produce comparable microbial or metabolic responses across populations. Cross-cultural validation of PHD indices and multiethnic intervention studies integrating microbiome and metabolomic outcomes are therefore needed to assess the generalizability of the proposed PHD–microbiome–healthy aging axis.

Another important priority is the identification of robust microbiome biomarkers of healthy aging. Although numerous microbial taxa, metabolites, and functional pathways have been associated with longevity and reduced disease risk, no validated microbiome signatures are currently available to predict healthspan, frailty, or responsiveness to dietary interventions. Establishing reliable biomarkers could improve risk stratification, preventive strategies, and personalized nutritional counseling.

Recent advances in metagenomics, metatranscriptomics, metabolomics, proteomics, and related multi-omics technologies are transforming nutritional aging research. Future studies should move beyond descriptive analyses of microbial composition toward integrated characterization of microbial function, metabolic activity, host–microbiome interactions, and validated biomarkers of biological aging, including epigenetic aging measures [354]. Incorporating relevant environmental and lifestyle exposures—including medication use, physical activity, sleep, psychosocial stress, socioeconomic conditions, and environmental pollutants—may provide a more comprehensive understanding of the factors shaping individual aging trajectories [354,355].

Artificial intelligence and machine-learning approaches may assist in integrating complex datasets, including microbiome sequencing, dietary intake, metabolomics, wearable sensor data, and electronic health records. Their clinical value, however, will depend on external validation, transparent analytical methods, and the availability of standardized, high-quality datasets.

Clinical translation will require harmonized dietary assessment, microbiome sampling, sequencing, bioinformatic analysis, and metabolomic profiling, together with outcomes that capture functional aging rather than disease incidence alone.

Future intervention studies should also investigate microbiome-targeted approaches that complement dietary modification. Carefully selected probiotics, prebiotics, synbiotics, postbiotics, fermented foods, and next-generation beneficial microorganisms may provide additional opportunities to enhance microbial function, although their long-term efficacy and generalizability require confirmation in adequately powered human trials.

Longitudinal studies integrating microbiome, metabolomic, dietary, and functional assessments are needed to distinguish causal mediators from response biomarkers and secondary correlates of healthier dietary patterns.

## 12. Limitations

Several limitations of the current evidence should be acknowledged. Direct human studies simultaneously evaluating adherence to the PHD, longitudinal gut microbiome changes, and clinically relevant healthy aging outcomes remain scarce. Much of the proposed mechanistic framework is therefore based on experimental studies, selected microbial metabolites, and indirect human evidence from related plant-rich dietary patterns. Considerable heterogeneity in dietary assessment, microbiome methodology, population characteristics, medication use, and outcome definitions further limits direct comparison across studies. As a narrative review, the present synthesis may also be subject to selection and interpretative bias despite the use of a structured literature search.

## 13. Conclusions

The available evidence identifies the gut microbiome as an important, but not exclusive, biological interface between PHD-consistent dietary patterns and healthy aging. Fiber fermentation, microbial transformation of polyphenols and tryptophan, bile acid signaling, and reduced endotoxin exposure provide plausible pathways connecting dietary composition with immune, metabolic, and cellular homeostasis. However, direct evidence demonstrating that microbiome changes mediate the long-term effects of the PHD on human healthspan remains limited. Resolving these uncertainties will be essential to determine whether microbiome-informed dietary strategies provide benefits beyond established nutritional recommendations.

## Figures and Tables

**Table 1:** Microbiome-mediated mechanisms of the major components of the Planetary Health Diet.

PHD Component	Primary Microbiome Effect	Major Microbial Metabolites	Principal Host Pathways	Physiological Outcome
Plant diversity	Increased microbial diversity and ecosystem resilience	SCFAs	Immune and metabolic homeostasis	Healthy microbiome aging
Dietary fiber	Enhanced microbial fermentation	Butyrate, propionate, acetate	AMPK–mTOR–autophagy	Cellular homeostasis
Polyphenols	Microbial biotransformation	Urolithins, phenolic acids	SIRT1–FOXO	Mitochondrial homeostasis
Legumes	Enhanced prebiotic fermentation	SCFAs	GLP-1 signaling	Improved metabolic regulation
Nuts and seeds	Enrichment of *Akkermansia muciniphila*	Secondary bile acids	FXR/TGR5 signaling	Metabolic and immune homeostasis
Fermented foods	Exposure to live microorganisms and fermentation-derived metabolites	Postbiotics, SCFAs	Mucosal immune regulation	Improved intestinal barrier integrity
Reduced red and processed meat	Reduced microbial TMA production	↓ TMAO	Reduced inflammatory signaling	Improved cardiometabolic health
Healthy fats	Remodeling of gut microbial ecology	SCFAs, lipid-derived metabolites	Mitochondrial homeostasis	Enhanced cellular resilience

Abbreviations: PHD, Planetary Health Diet; SCFAs, short-chain fatty acids; AMPK, AMP-activated protein kinase; mTOR, mechanistic target of rapamycin; SIRT1, sirtuin 1; FOXO, Forkhead box O transcription factors; GLP-1, glucagon-like peptide-1; FXR, farnesoid X receptor; TGR5, G protein-coupled bile acid receptor 1; TMAO, trimethylamine *N*-oxide. ↓ indicates a decrease.

**Table 2:** Proposed microbiome-mediated regulation of major longevity-associated signaling pathways and its relevance to healthy aging.

Longevity-Associated Pathway	Major Microbiome-Derived Regulators	Key Cellular Functions	Potential Contribution to Healthy Aging	Predominant Evidence Base
AMPK	Butyrate, propionate, secondary bile acids	Energy sensing; fatty acid oxidation; autophagy; mitochondrial homeostasis	Improved metabolic flexibility, reduced inflammation, and greater cellular resilience	Mainly animal and in vitro studies, with indirect human evidence from fiber-rich and plant-based dietary interventions; limited direct PHD-specific evidence
mTOR	SCFAs; altered nutrient availability; microbiome-derived metabolites	Nutrient sensing; protein synthesis; autophagy regulation	Improved proteostasis and maintenance of tissue homeostasis	Mainly animal and in vitro evidence, with indirect human evidence from protein-composition and plant-rich dietary studies; no direct PHD-specific microbiome evidence
IGF-1	SCFAs; microbiome-mediated nutrient metabolism	Insulin/IGF-1 signaling; anabolic regulation	Improved metabolic regulation and balance between growth and cellular repair	Human observational evidence, complemented by animal and mechanistic studies; limited direct microbiome and PHD-specific evidence
SIRT1	Butyrate, urolithin A, phenolic metabolites	NAD^+^-dependent signaling; mitochondrial quality control; stress adaptation; anti-inflammatory regulation	Improved mitochondrial function, metabolic homeostasis, and cellular resilience	Mainly animal and in vitro studies, with limited human intervention evidence for selected metabolites; no direct PHD-specific evidence
FOXO	Indole derivatives, SCFAs, polyphenol-derived metabolites	Oxidative stress resistance; DNA repair; apoptosis; cellular stress responses	Maintenance of cellular homeostasis and resilience to age-related stress	Mainly animal and in vitro evidence; human evidence is largely indirect and based on dietary, metabolic, or biomarker associations
Autophagy	Butyrate, urolithin A, secondary bile acids	Removal of damaged proteins and organelles; mitophagy; proteostasis	Preservation of cellular function and physiological resilience	Mainly animal and in vitro evidence, with limited human studies of selected microbial metabolites; no direct demonstration of PHD-induced autophagy through the gut microbiome

Abbreviations: AMPK, AMP-activated protein kinase; mTOR, mechanistic target of rapamycin; SCFAs, short-chain fatty acids; IGF-1, insulin-like growth factor 1; SIRT1, sirtuin 1; FOXO, Forkhead box O transcription factors; NAD^+^, nicotinamide adenine dinucleotide; DNA, deoxyribonucleic acid; PHD, Planetary Health Diet. Note: The evidence base indicates the predominant type of evidence supporting each proposed microbiome–signaling relationship. Direct human evidence specifically linking PHD-induced microbiome changes to downstream longevity-associated signaling pathways remains limited; most relationships are supported by experimental studies and indirect human evidence from related plant-rich dietary patterns, individual dietary components, or selected microbial metabolites.

**Table 3:** Organ-specific effects mediated by the Planetary Health Diet–gut microbiome axis.

Organ System	Major Microbiota-Derived Mediators	Principal Molecular Targets	Major Aging-Related Outcomes
Brain (gut–brain axis) [301,302,303]	SCFAs, indole derivatives, tryptophan metabolites	Microglia, BDNF, vagal signaling, neuroinflammation	Cognitive aging, Alzheimer’s disease, Parkinson’s disease
Cardiovascular system [304,305,306,307]	SCFAs, TMAO, secondary bile acids	Endothelial function, blood pressure regulation, vascular inflammation	Atherosclerosis, heart failure, healthy vascular aging
Skeletal muscle (gut–muscle axis) [308,309,310,311,312]	SCFAs, amino acid metabolites	Mitochondrial function, protein synthesis, muscle metabolism	Frailty, sarcopenia, physical performance
Immune system [264,313,314]	SCFAs, indoles, microbial antigens	Treg differentiation, IL-10, IL-6, TNF-α, immune homeostasis	Immunosenescence, inflammaging, healthy immune aging
Liver (gut–liver axis) [104,315,316,317,318]	Secondary bile acids, SCFAs	FXR, TGR5, lipid metabolism, glucose homeostasis	MASLD, metabolic dysfunction, healthy liver aging
Bone (gut–bone axis) [319,320,321,322,323]	SCFAs, bile acids	Calcium absorption, osteoblast activity, osteoclast regulation	Osteoporosis prevention, healthy skeletal aging

Abbreviations: BDNF, brain-derived neurotrophic factor; FXR, farnesoid X receptor; IL, interleukin; MASLD, metabolic dysfunction-associated steatotic liver disease; SCFAs, short-chain fatty acids; TGR5, Takeda G protein-coupled bile acid receptor 5 (G protein-coupled bile acid receptor 1); TMAO, trimethylamine *N*-oxide; TNF-α, tumor necrosis factor alpha; Treg, regulatory T cells.

**Table 4:** Representative human evidence on the Planetary Health Diet and related dietary patterns, distinguishing direct healthy aging outcomes from age-related disease and intermediate health outcomes.

Study	Population	Study Design	Dietary Pattern	Primary Outcome	Main Findings
Planetary Health Diet cohorts					
Wang et al., 2025 [198]	NHANES (42,947), UK Biobank (125,372), meta-analysis (37 cohorts; 3.24 million participants)	Prospective cohorts + meta-analysis	Planetary Health Diet	Mortality and chronic diseases	Greater adherence to the Planetary Health Diet was associated with lower all-cause mortality and reduced risks of cardiovascular disease, cancer, stroke, and type 2 diabetes.
Bui et al., 2024 [18]	NHS (66,692 women), NHS II (92,438 women), HPFS (47,274 men); 206,404 participants	Three prospective cohorts	Planetary Health Diet Index (PHDI)	All-cause and cause-specific mortality	Higher PHDI adherence was associated with 23% lower all-cause mortality and lower cardiovascular, cancer, respiratory, neurodegenerative, and infectious disease mortality, together with reduced environmental impacts.
Karavasiloglou et al., 2023 [324]	UK Biobank, 473,836 adults	Prospective cohort	EAT-Lancet reference diet	Incident cancer, major cardiovascular events, all-cause mortality	Higher adherence was associated with 9% lower incident cancer risk and 10% lower all-cause mortality, while no significant association was observed with major cardiovascular events.
Guzmán-Castellanos et al., 2024 [325]	18,656 Spanish adults (SUN cohort)	Prospective cohort (median follow-up: 11.5 years)	Planetary Health Diet Index	Incident cardiovascular disease	Higher adherence to the Planetary Health Diet showed a non-significant trend toward lower CVD risk, but no statistically significant association was observed (HR 0.77, 95% CI 0.51–1.18).
Knuppel et al., 2019 (EPIC-Oxford) [326]	46,069 UK adults	Prospective cohort (up to 23.6 years follow-up)	EAT-Lancet reference diet score	Ischaemic heart disease, stroke, type 2 diabetes, all-cause mortality	Greater adherence to the EAT-Lancet diet was associated with a lower risk of ischaemic heart disease (−28%) and type 2 diabetes (−59%), with no significant associations for stroke or all-cause mortality.
Healthy aging cohorts					
Tessier et al., 2025 [327]	105,015 US adults (NHS, HPFS)	Prospective cohort, 30-year follow-up	AHEI, aMED, DASH, MIND, hPDI, PHDI, rEDIH, rEDIP	Healthy aging	Greater long-term adherence to healthy dietary patterns was consistently associated with higher odds of healthy aging. AHEI showed the strongest overall association, while higher ultra-processed food intake was inversely associated with healthy aging.
Maroto-Rodriguez et al., 2025 [328]	UK Biobank, 19,505 middle-aged and older adults	Prospective cohort (median follow-up: 6.25 years)	Planetary Health Diet Index (PHDI)	Healthy aging (intrinsic capacity and frailty)	Higher PHDI adherence was associated with greater intrinsic capacity and a lower risk of frailty, supporting the role of the Planetary Health Diet in promoting healthy aging.
Gómez-Cao et al., 2025 [194]	Seniors-ENRICA-1 and -2 cohorts, Spain (*n* = 2998 adults aged ≥ 60 years)	Prospective cohort (median follow-up: 2.6 years)	Planetary Health Diet Index (PHDI)	Intrinsic capacity	Higher adherence to the PHDI was associated with better preservation of intrinsic capacity, particularly hearing function, supporting a beneficial role of the Planetary Health Diet in healthy aging.
Related healthy aging cohorts					
Kim et al., 2024 [329]	Korean Frailty and Aging Cohort Study, 665 community-dwelling adults aged 70–84 years	6-year prospective cohort	Healthy dietary patterns (variety of healthy foods vs. rice-based patterns)	Intrinsic capacity	Greater adherence to a healthy, diverse dietary pattern was associated with better preservation of intrinsic capacity, particularly psychological function, supporting the role of diet quality in healthy aging.
Gopinath et al., 2016 [330]	Blue Mountains Eye Study (Australia); 1609 adults aged ≥ 49 years	10-year prospective cohort	Dietary carbohydrate quality (fiber, glycemic index, glycemic load)	Successful aging	Higher dietary fiber intake, particularly from whole grains and fruits, was independently associated with greater odds of successful aging over 10 years, whereas glycemic index, glycemic load, and total carbohydrate intake were not significantly associated with successful aging.
Wang et al., 2023 (Million Veteran Program) [331]	315,919 US veterans aged 19–104 years	Prospective cohort	Overall, healthful, and unhealthful plant-based diet indices (PDI, hPDI, uPDI)	All-cause and cause-specific mortality	Greater adherence to overall and healthful plant-based diets was associated with lower all-cause, cardiovascular, and cancer mortality, whereas greater adherence to an unhealthful plant-based diet was associated with increased mortality risk.
Wang et al., 2018 [332]	10,210 older adults from five prospective cohorts (six studies overall)	Systematic review and meta-analysis	Mediterranean diet	Incident frailty	Higher adherence to the Mediterranean diet was associated with a 44% lower risk of incident frailty (RR 0.56, 95% CI 0.36–0.89), with an even greater reduction among Western populations, supporting Mediterranean-style dietary patterns for healthy aging and frailty prevention.
Mediterranean diet RCTs (indirect supportive evidence)					
Estruch et al., 2018 (PREDIMED) [333]	7447 Spanish adults at high cardiovascular risk	Randomized controlled trial (median follow-up: 4.8 years)	Mediterranean diet supplemented with extra-virgin olive oil or nuts	Major cardiovascular events	Compared with a low-fat diet, Mediterranean diet interventions reduced the incidence of major cardiovascular events by approximately 30%, providing robust evidence for the cardiovascular benefits of a Mediterranean/PHD-like dietary pattern.
Delgado-Lista et al., 2022 (CORDIOPREV) [334]	1002 adults (20–75 years) with established coronary heart disease, Spain	Randomized controlled trial (median follow-up: 7 years)	Mediterranean diet vs. low-fat diet	Major cardiovascular events (secondary prevention)	Compared with a low-fat diet, the Mediterranean diet reduced the risk of recurrent major cardiovascular events by approximately 28% (adjusted HR 0.72–0.75), demonstrating superior long-term efficacy for secondary cardiovascular prevention.
Salas-Salvadó et al., 2011 (PREDIMED-Reus) [335]	418 adults at high cardiovascular risk without diabetes	Randomized controlled trial (4-year follow-up)	Mediterranean diet + EVOO or nuts vs. low-fat diet	Incident type 2 diabetes	Mediterranean diet reduced incident type 2 diabetes by 52% versus a low-fat diet, independent of weight loss or physical activity, supporting its role in diabetes prevention.
Martínez-Lapiscina et al., 2013 (PREDIMED-NAVARRA) [336]	522 older adults at high cardiovascular risk	Randomized controlled trial (6.5-year follow-up)	Mediterranean diet supplemented with extra-virgin olive oil or nuts vs. low-fat diet	Global cognitive function	Mediterranean diet significantly improved global cognitive performance (MMSE and Clock Drawing Test) compared with a low-fat diet, supporting long-term cognitive benefits.
Valls-Pedret et al., 2015 (PREDIMED) [337]	447 older adults at high cardiovascular risk	Randomized clinical trial (median follow-up 4.1 years)	Mediterranean diet + extra-virgin olive oil or nuts vs. low-fat diet	Cognitive function	Mediterranean diet improved memory, executive function, and global cognition, attenuating age-related cognitive decline compared with the control diet.
Evidence synthesis					
Dinu et al., 2018 [338]	>12.8 million participants from observational studies and randomized controlled trials	Umbrella review of 29 meta-analyses	Mediterranean diet	Multiple healthy aging outcomes	High adherence to the Mediterranean diet was consistently associated with lower all-cause mortality, cardiovascular disease, coronary heart disease, myocardial infarction, overall cancer incidence, neurodegenerative diseases, and type 2 diabetes, providing robust evidence for broad health benefits.
Martínez-González et al., 2015 (PREDIMED) [339]	PREDIMED trial (7447 high-risk adults) and supporting prospective cohorts	Narrative review	Mediterranean diet	Cardiometabolic outcomes	Summarized evidence from the PREDIMED trial and large prospective cohorts demonstrating that Mediterranean dietary patterns reduce major cardiovascular events, type 2 diabetes, and cardiometabolic risk through anti-inflammatory and antioxidant mechanisms, supporting their role as a sustainable healthy aging dietary model.

Abbreviations: AHEI, Alternative Healthy Eating Index; aMED, Alternate Mediterranean Diet Score; CI, confidence interval; CORDIOPREV, Coronary Diet Intervention with Olive Oil and Cardiovascular Prevention Study; CVD, cardiovascular disease; DASH, Dietary Approaches to Stop Hypertension; EAT-Lancet, EAT-Lancet Commission healthy reference diet; ENRICA, Study on Nutrition and Cardiovascular Risk in Spain (Estudio sobre Nutrición y Riesgo Cardiovascular); EPIC, European Prospective Investigation into Cancer and Nutrition; EVOO, extra-virgin olive oil; HPFS, Health Professionals Follow-up Study; HR, hazard ratio; hPDI, healthful Plant-Based Diet Index; MIND, Mediterranean-DASH Intervention for Neurodegenerative Delay; MMSE, Mini-Mental State Examination; NHANES, National Health and Nutrition Examination Survey; NHS, Nurses’ Health Study; NHS II, Nurses’ Health Study II; PDI, Plant-Based Diet Index; PHD, Planetary Health Diet; PHDI, Planetary Health Diet Index; PREDIMED, Prevención con Dieta Mediterránea; RCT, randomized controlled trial; rEDIH, reverse Empirical Dietary Index for Hyperinsulinemia; rEDIP, reverse Empirical Dietary Inflammatory Pattern; RR, relative risk; SUN, Seguimiento Universidad de Navarra; uPDI, unhealthful Plant-Based Diet Index; UK, United Kingdom. Note: Mediterranean diet studies are considered supportive but indirect evidence and not direct evaluations of the PHD. Functional capacity, intrinsic capacity, frailty, healthspan, and longevity are treated as direct aging-related outcomes, whereas chronic disease, cardiometabolic outcomes, and biomarkers are considered intermediate outcomes relevant to, but not equivalent to, healthy aging.

## Data Availability

No new data were created or analyzed in this study. Data sharing is not applicable to this article.
